# Applying the “positive predictive value–recall diagram” to monitor performance and provide recommendations for screening radiologists

**DOI:** 10.1007/s00330-025-11978-3

**Published:** 2025-09-04

**Authors:** Tanya D. Geertse, Eric Tetteroo, Maartje J. A. Smid-Geirnaerdt, Lucien E. M. Duijm, Ruud M. Pijnappel, Daniëlle van der Waal, Mireille J. M. Broeders

**Affiliations:** 1 Dutch Expert Centre for Screening (LRCB), Nijmegen, The Netherlands; 2 Department of Radiology, Amphia Hospital, Breda, The Netherlands; 3 Department of Radiology, Canisius Wilhelmina Hospital, Nijmegen, The Netherlands; 4 Department of Radiology, University Medical Centre Utrecht, Utrecht University, Utrecht, The Netherlands; 5 IQ Health Science Department, Radboud University Medical Center, Nijmegen, The Netherlands

**Keywords:** Breast neoplasms, Early detection of cancer, Quality assurance, Medical audit

## Abstract

**Objectives:**

To evaluate the suitability of “positive predictive value–recall” (PPV-recall) diagrams for monitoring performance and providing recommendations for groups of radiologists (RUs or reading units) in breast cancer screening.

**Materials and methods:**

This retrospective study used datasets from triennial quality assurance audits within the Dutch screening programme. The recall rate (RR), cancer detection rate (CDR), and PPV between 2010 and 2019 were plotted in PPV-recall diagrams separately for initial and subsequent screening. Using PPV-recall diagrams per year we compared variations in performance of the RUs within the screening programme. Each group’s screening behaviour characteristics were evaluated over time with RU-specific PPV-recall diagrams and related audit recommendations.

**Results:**

The dataset comprised the aggregated results of 779,887 initial and 6,021,598 subsequent screenings read by 12 RUs between 2010 and 2019. The PPV-recall diagrams showed substantial variations in the individual RU performance over time, with PPVs ranging between 4.9 and 23.7% for initial and 21.2–54.3% for subsequent screening. Target values were less often met for initial (2010: 0 RUs; 2019: 5 RUs) than for subsequent screening (2010: 8 RUs; 2019: 10 RUs), resulting in more recommendations regarding initial screening (24 versus 13). All recommendations focused on adjusting RR, which often (17 out of 24) changed in the recommended direction, though not always sufficient to meet target values.

**Conclusion:**

PPV-recall diagrams offer valuable insights into variations and interrelationships between screening outcomes, helping the audit team in providing recommendations for improvement. However, feedback based on these diagrams alone may not always be sufficient for individual radiologists to achieve these improvements.

**Key Points:**

***Question*** *Can positive predictive value (PPV)–recall diagrams help audit teams provide recommendations to radiologists to enhance their reading performance in a breast cancer screening programme?*

***Findings**** PPV-recall diagrams help audit teams identify screening outcome variation. Recall rates often changed in the desired direction after recommendations, but did not always meet targets*.

***Clinical relevance**** Incorporating PPV-recall diagrams into quality assurance audits in breast cancer screening can support audit teams to provide recommendations to radiologists to maximise cancer detection and minimise false positives. Radiologists may need additional individual feedback to optimally achieve these improvements*.

**Graphical Abstract:**

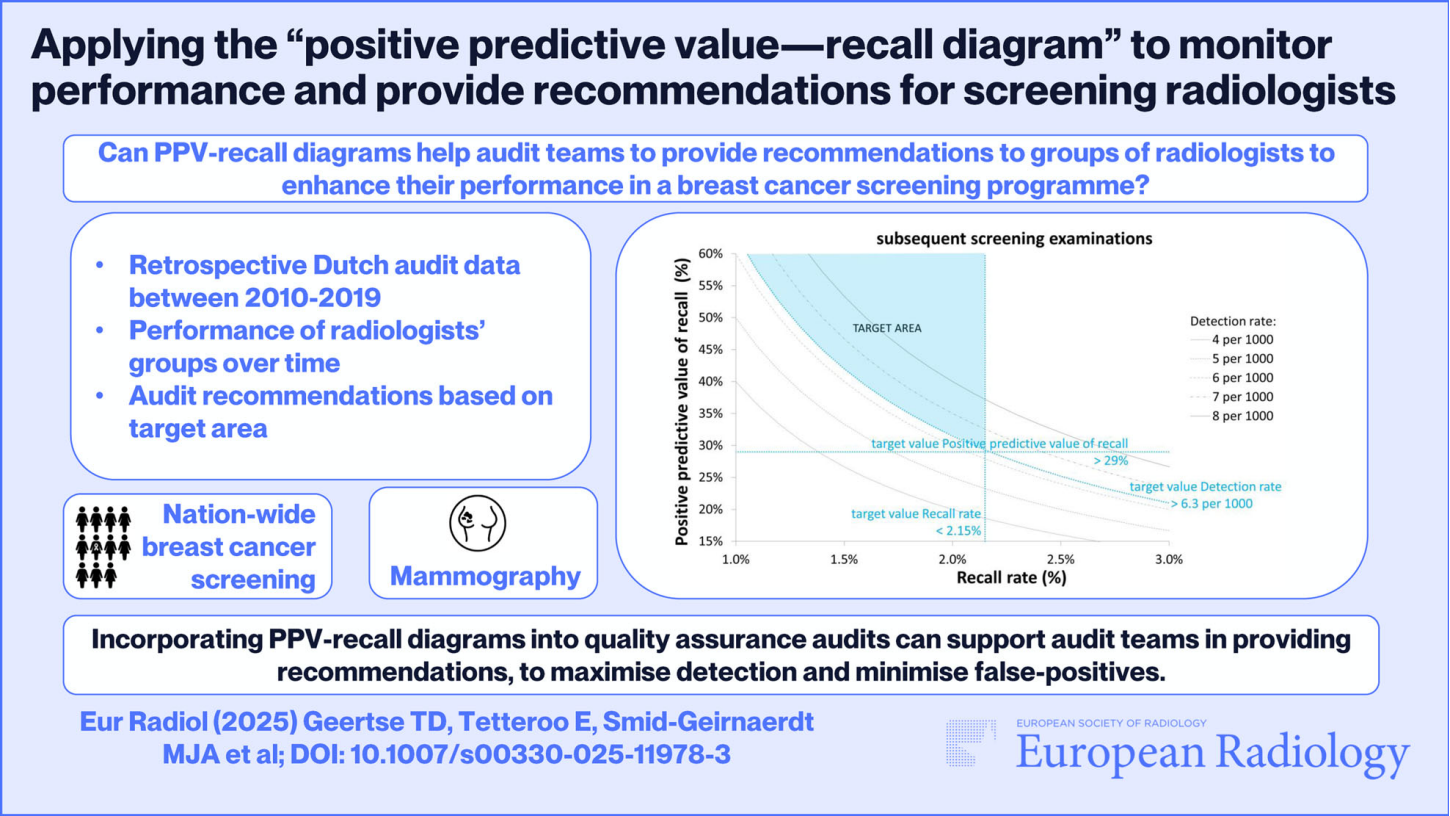

## Introduction

Breast cancer screening can prevent breast cancer-related death through early detection and treatment; however, it also inflicts harms such as overdiagnosis and false-positive screening results [1, 2]. Quality assurance (QA) programmes, including quality control of equipment, training and accreditation of professionals, and monitoring of screening outcomes, should ensure a favourable balance between the benefits and harms and guarantee a high level of quality within the screening programme [3–5].

For monitoring screening outcomes, an effective method is needed to provide insight into the reading performance of screening radiologists, to identify areas for improvement and pinpoint (groups of) radiologists who may require additional training. Recall rate (RR), cancer detection rate (CDR), and positive predictive value of recall (PPV) are key parameters for this purpose, which are best monitored combined, as they are interrelated [6]. In 2001, Blanks introduced the PPV-recall diagram as a method for monitoring screening outcomes [7]. In a PPV-recall diagram, the PPV is plotted against the RR, with the CDR shown as “isobars” in the graph, which visualises the interrelationship between these three screening outcomes [7]. The diagram may also include target values and/or a national average of these screening outcomes.

In the Netherlands, the reading performance of the screening radiologists has been monitored at the group level, with a group referred to as a reading unit (RU), during triennial audits. As part of the audit, the PPV-recall diagrams for the RUs have been reviewed for several years [8]. The effectiveness of using the PPV-recall diagram to assess quality, identify areas for improvement and formulate recommendations has not been fully established.

Therefore, the aim of this study was to retrospectively evaluate the suitability of PPV-recall diagrams for monitoring reading performance and providing recommendations for RUs in a breast cancer screening programme.

## Materials and methods

This retrospective descriptive study was performed under the national permit for breast cancer screening issued by the Dutch Ministry of Health, Welfare and Sport, which is equivalent to approval by a local institutional review board.

### Screening procedure

The Dutch breast cancer screening programme is centrally organised by the national screening organisation, which manages all screening units and RUs. Biennially, women aged 50–74 years are invited for a screening examination, performed by radiographers specialised in mammography. The standard examination is two-view mammography (mediolateral oblique view and craniocaudal view). Initially, screen-film mammography was used, with mammography systems from different manufacturers. In 2008, the transition to digital mammography (Lorad Selenia, Hologic) began and was completed by June 2010.

All mammograms are independently read by two certified screening radiologists. Discrepancies are solved by consensus or arbitration by a third reader, and prior examinations are available for comparison in subsequent screening. Women with suspicious findings are recalled to a hospital for further assessment.

### Audits

**Fig. 1:**
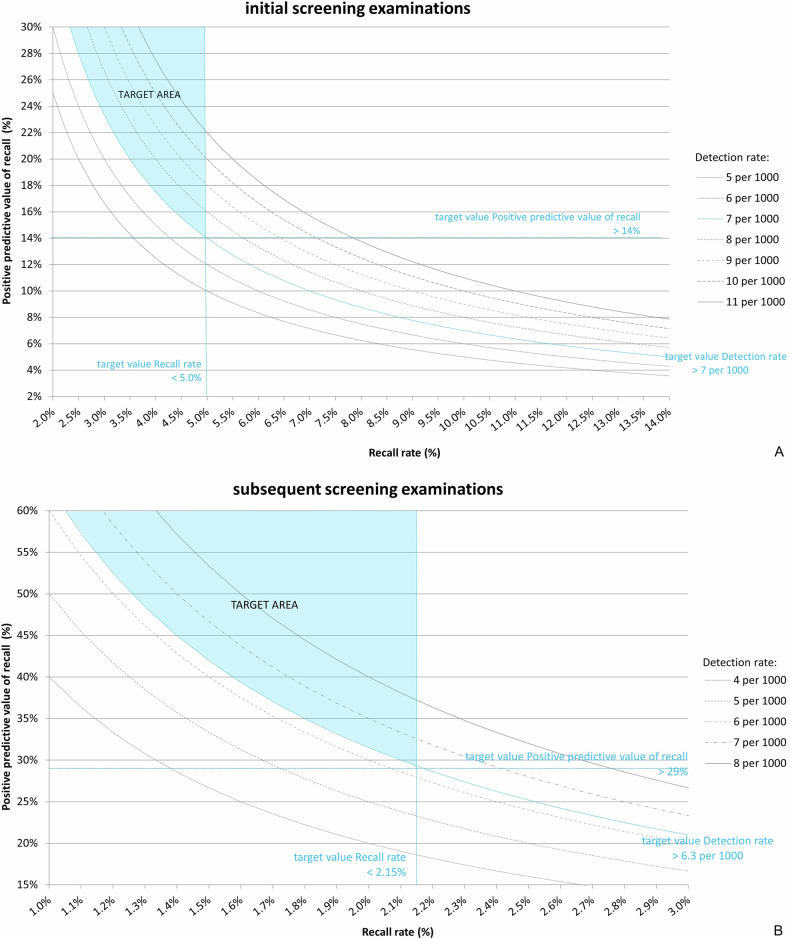
The PPV-recall diagram, showing positive predictive value of recall (PPV) versus recall rate (RR), with cancer detection rate (CDR) presented as isobars for initial screening (**A**) and subsequent screening (**B**). The “target area”, where all target values are met, is shaded in blue. Screening outcomes outside this area deviate from the targets, and specific recommendations should be issued

Triennial audits are conducted by the Dutch Expert Centre for Screening (LRCB) following a fixed protocol, described in detail in a previous study [8]. In summary, the screening organisation provides a dataset for each audit, divided into initial and subsequent screening outcomes, covering all examinations in a 4-year period. The oldest year usually overlaps with the previous audit, as audits are conducted approximately every 3 years. For each audit, the mean screening outcomes over the 4-year period are compared to target values (Table 1) and mean national values [9, 10], using tables and graphs, including the PPV-recall diagram. If the RR, PPV or CDR do not meet the target value, and thus the RU performs outside the “target area” in the PPV-recall diagram (see Fig. 1), the audit team recommends improvements to the RU (audit recommendation).

**Table 1:** Target values set for the Dutch breast cancer screening programme

Performance measure	Until July 2016	From July 2016
Initial screening:		
Recall rate (%)	2.5–3.5^a^	< 5.0
Detection rate (per 1000 screens)	> 5.5	> 7
Positive predictive value of recall (%)	> 16	> 14
Subsequent screening:		
Recall rate (%)	1.3–2.0^a^	< 2.15
Detection rate (per 1000 screens)	> 4.5	> 6.3
Positive predictive value of recall (%)	> 22.5	> 29

^a^ Until July 2016, for the recall rate, a target range was set instead of a target value

After the transition to digital mammography in 2010, RRs increased considerably [11]. This led to the adjustment of target values in July 2016 (Table 1), based on an advisory report from the LRCB to the policymakers [12].

### Data collection

**Fig. 2:**
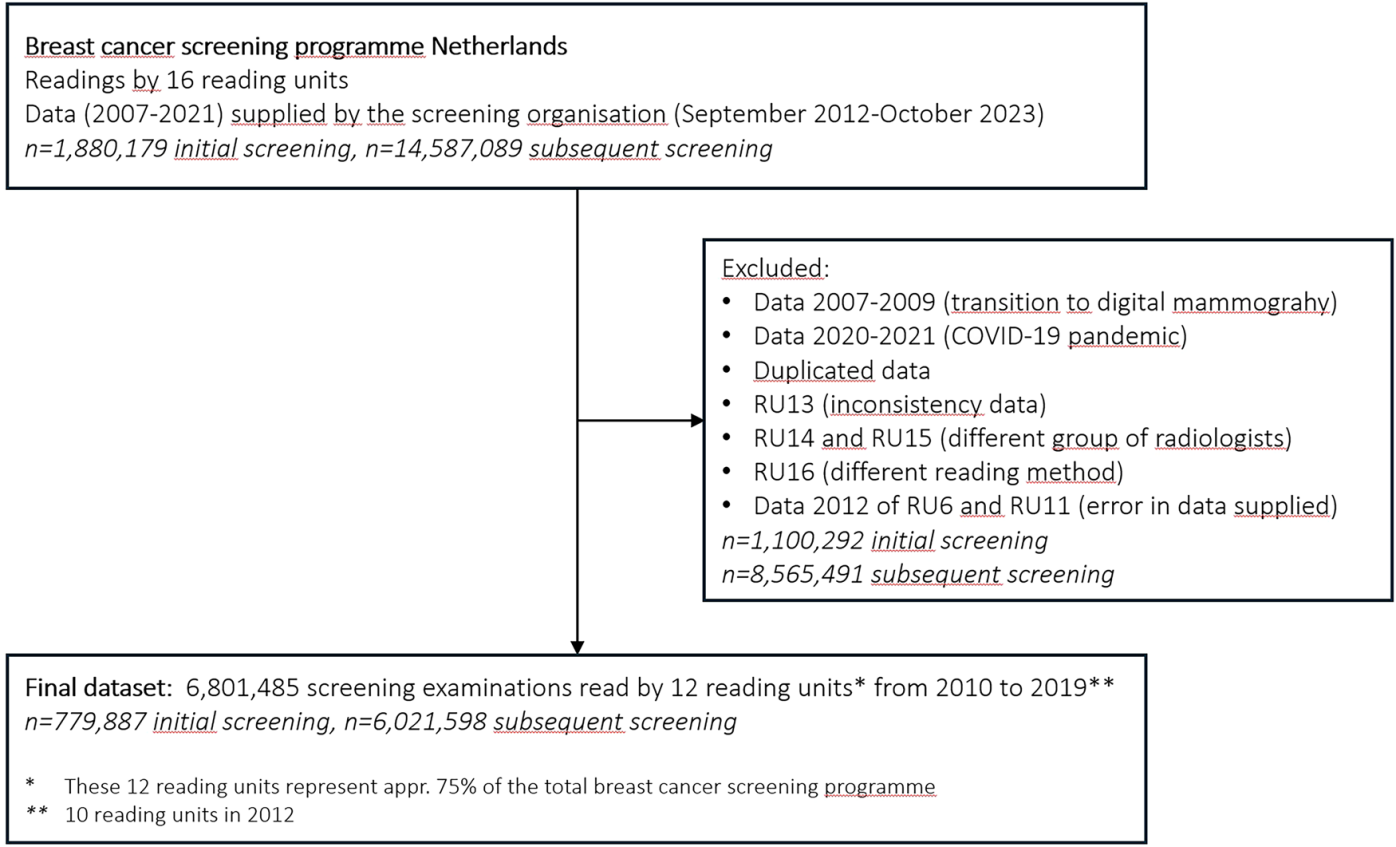
Flowchart to describe the selection of study data

Data for this study were supplied by the screening organisation for audits conducted between September 2012 and October 2023, monitoring reading performance from 2007 to 2021 (Fig. 2). Data were extracted on the number of screenings, recalls, and screen-detected carcinomas (in situ and invasive) per year, for both initial and subsequent screening. Only digital mammography data from 2010 to 2019 were used. Data from 2007 to 2009 were excluded due to the transition from analogue to digital mammography, and data from 2020 to 2021 were excluded due to the impact of the COVID-19 pandemic on the screening programme. In case of duplicate data, due to the triennial audit cycle and overlapping 4-year periods (see “Audits”), only the most recent submissions were included. For inclusion, the data from an RU had to be complete and consistent, there could not have been a complete turnover of the radiologist team, and the RU had to adhere to the standard reading method. Otherwise, the RU was excluded (see Fig. 2). Audit reports were also available, describing audit outcomes and recommendations given to the RUs.

### Data analysis

The screening outcomes RR, CDR, and PPV were calculated with 95% confidence intervals (95% CI), presented separately for initial and subsequent screening. The 95% CIs were calculated using the standard formula: $95 \% {CI}=P\pm 1.96* \scriptstyle\sqrt{\frac{P* (1-P)}{N}}$, where *P* is the observed proportion and *N* is the number of screenings used to calculate the proportion. Furthermore, we calculated the median with interquartile range (IQR) for the RR, CDR, and PPV for each year.

The screening outcomes were graphically depicted in PPV-recall diagrams for both initial and subsequent screening, for each individual year within the study period (2010–2019). Each diagram contains data points for the 12 RUs, showing how they performed relative to each other. In addition, PPV-recall diagrams were plotted for each individual RU over a 10-year period. In these diagrams, audit years and recommendations were also indicated, enabling assessment of performance changes following audits, thus focusing more on the screening behaviour characteristics of each RU.

For all recommendations given by the audit team (based on the mean RR during the evaluation period), absolute changes in RR were assessed over the 3 years following the audit. We used the RR from the last year of the evaluation period as the ‘baseline’ for initial and subsequent screening. If the audit took place between 1 January and 30 June, the year in which the audit took place was also considered to be the first year after the audit. Changes in RR could not be assessed if the first year after the audit fell outside the study period (2020 or later).

## Results

The dataset supplied by the screening organisation for the audits conducted between September 2012 and October 2023 consisted of the aggregated screening results of 16,467,268 mammography screenings (1,880,179 initial screenings and 14,587,089 subsequent screenings), read by 16 RUs. In total, 9,665,783 screenings were excluded (1,100,292 initial screenings and 8,565,491 subsequent screenings). Our final dataset consisted of the aggregated screening results of 6,801,485 digital mammography screenings read by 12 RUs from 2010 to 2019 (Fig. 2). The aggregated results per year are presented in Tables 2 and 3, for initial (*n* = 779,887) and subsequent screening (*n* = 6,021,598), respectively.

**Table 2:** Aggregated results per year of all 12 reading units for recall rate, PPV of recall and detection rate for the initial screening examinations

Year (*n* = 12)	Total initial screening examinations	Total recalls	Total screen-detected cancers	Recall rate %, median (IQR), range	PPV of recall %, median (IQR), range	Detection rate per 1000, median (IQR), range
2010	74,399	3719	522	4.9 (4.1–6.1), 3.8–9.3	13.3 (10.1–17.6), 8.8–21.8	6.9 (5.9–7.8), 4.9–10.0
2011	80,504	4103	586	5.3 (4.3–6.3), 3.7–7.3	14.3 (10.4–19.1), 7.4–21.5	7.9 (5.5–8.4), 5.2–8.8
2012^a^	74,195	4234	597	5.1 (4.4–8.5), 4.1–9.5	15.3 (10.0–19.2), 7.9–20.5	8.1 (7.8–8.6), 6.8–9.5
2013	78,939	5162	689	6.1 (4.9–9.4), 4.1–11.5	11.8 (9.5–18.7), 7.4–21.6	8.7 (7.8–10.0), 5.0–10.9
2014	81,557	4921	634	5.5 (4.8–7.2), 3.6–12.4	13.1 (11.3–15.8), 4.9–23.7	7.8 (6.3–8.6), 5.1–10.6
2015	80,455	4837	674	6.8 (5.2–7.2), 3.1–9.0	14.4 (12.0–16.7), 6.4–22.7	8.5 (7.0–10.1), 5.8–10.7
2016	80,620	5187	743	7.0 (5.5–7.5), 3.2–9.3	13.2 (11.3–18.4), 10.4–23.2	9.3 (8.6–9.6), 7.3–10.6
2017	77,698	4924	640	6.2 (5.3–7.7), 3.8–9.1	13.4 (10.6–15.4), 9.1–18.3	8.1 (7.1–9.1), 5.9–9.7
2018	76,104	4509	620	5.6 (5.1–6.8), 4.7–7.4	14.3 (13.0–16.6), 9.0–18.0	8.5 (6.8–9.2), 6.7–9.4
2019	75,416	4721	657	5.9 (5.2–6.9), 4.4–8.4	15.1 (11.6–16.7), 9.4–18.1	8.5 (7.8–9.4), 5.6–10.5

*IQR* interquartile range, *PPV* positive predictive value

^a^ *n* = 10 reading units

**Table 3:** Aggregated results per year of all 12 reading units for recall rate, PPV of recall and detection rate for the subsequent screening examinations

Year (*n* = 12)	Total subsequent screening examinations	Total recalls	Total screen-detected cancers	Recall rate %, median (IQR), range	PPV of recall %, median (IQR), range	Detection rate per 1000, median (IQR), range
2010	543,843	9220	3207	1.6 (1.4–2.1), 1.1–2.4	34.2 (28.5–40.6), 24.4–51.8	6.0 (5.2–6.3), 4.1–6.7
2011	607,849	10,534	3603	1.8 (1.5–1.9), 1.3–2.4	33.4 (32.4–38.5), 23.4–43.9	6.1 (5.7–6.3), 4.9–6.7
2012^a^	557,550	10,190	3396	1.9 (1.6–2.0), 1.4–2.5	33.4 (27.4–37.8), 24.4–41.0	6.1 (5.8–6.3), 5.1–7.0
2013	620,961	12,389	3956	1.9 (1.8–2.4), 1.5–2.8	31.1 (26.8–34.3), 24.3–42.6	6.4 (6.0–6.7), 5.6–7.9
2014	611,087	11,704	3926	1.9 (1.6–2.2), 1.1–2.9	34.7 (29.4–38.2), 21.2–46.7	6.2 (5.9–6.9), 5.3–7.5
2015	632,228	11,542	4135	1.9 (1.7–2.0), 1.1–2.2	34.7 (31.9–38.4), 30.4–54.3	6.6 (6.1–6.8), 5.8–7.4
2016	638,783	12,041	4276	1.9 (1.6–2.1), 1.0–2.5	35.0 (31.6–38.1), 28.5–54.0	6.8 (5.7–7.1), 5.3–8.0
2017	639,283	11,661	4112	1.8 (1.6–1.9), 1.0–2.5	34.6 (32.4–39.1), 28.4–51.1	6.5 (6.0–6.8), 5.2–7.2
2018	610,791	10,975	3925	1.7 (1.5–2.0), 1.3–2.4	36.6 (31.2–44.4), 28.3–45.6	6.3 (6.0–7.0), 5.3–7.1
2019	559,223	10,824	3759	1.8 (1.7–2.1), 1.4–2.7	37.2 (34.3–38.8), 26.7–40.1	6.8 (6.3–7.1), 5.7–7.5

*IQR* interquartile range, *PPV* positive predictive value

^a^ *n* = 10 reading units

### Variations and trends in RU performance, for initial screening

For initial screening, the PPV-recall diagrams per year for 2010 to 2019 show substantial variations in performance among the individual RUs. The lowest PPV observed during the study period was 4.9% (2014, RU12) with a RR of 12.4% and a CDR of 6.1 per 1000 screenings. Conversely, the highest PPV was 23.7% (2014, RU2), based on a RR of 3.6% and a CDR of 8.6 per 1000 screenings. For overall performance across all units, the lowest median PPV was 11.8% (2013), and the highest median PPV was 15.3% (2012) (Table 2).

**Fig. 3:**
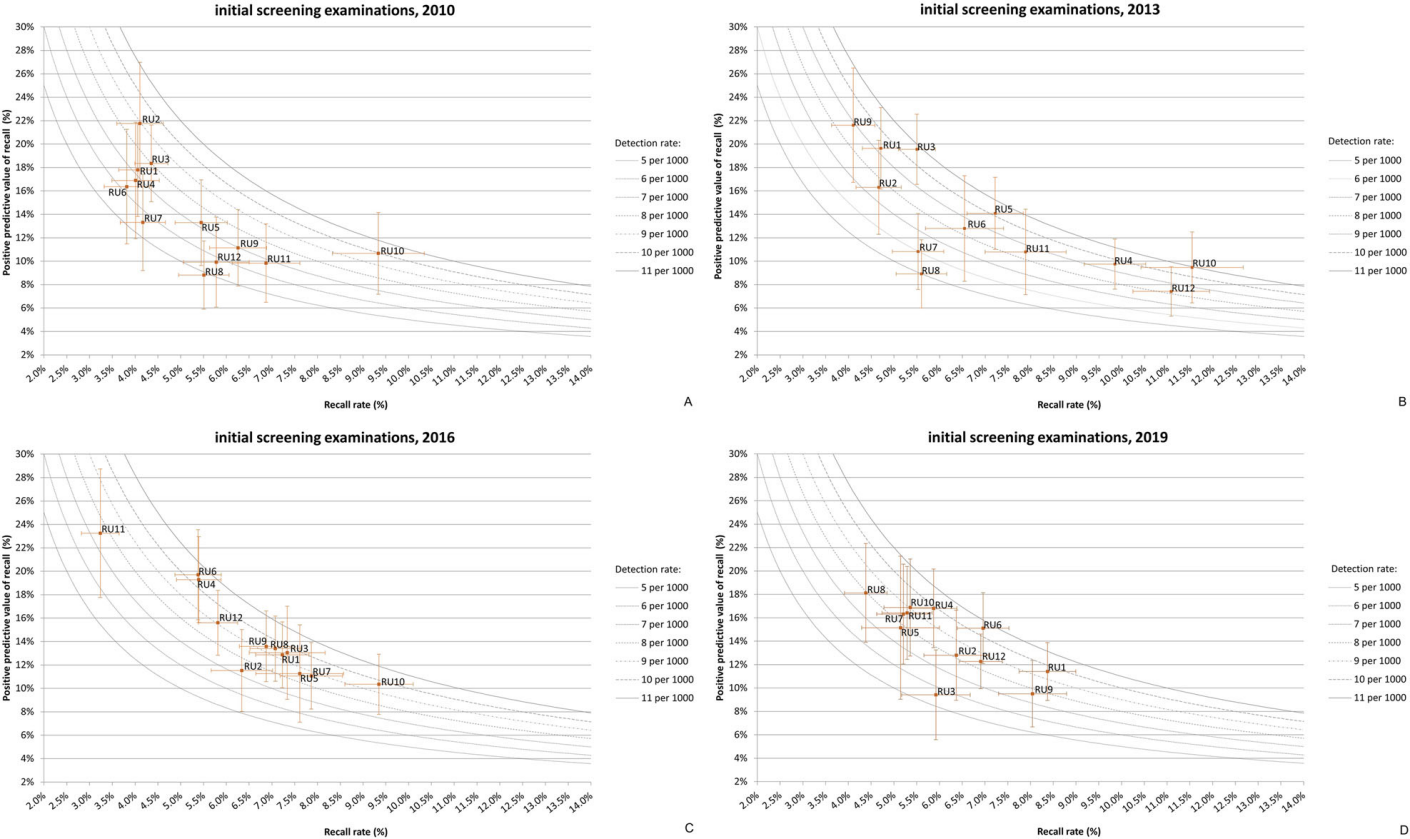
PPV-recall diagrams, with cancer detection rate (CDR) presented as isobars, for initial screening examinations for 2010 (**A**), 2013 (**B**), 2016 (**C**) and 2019 (**D**), showing how the 12 RUs performed relative to each other

In 2010 (Fig. 3A), following the transition to digital mammography, all RUs performed outside the “target area”. The “target area” refers to the range where all three target values were met. Specifically, five RUs (RU8-RU12) had a PPV below the target value (16%), and all RUs had a RR exceeding the target value (3.5%). The median RR was 4.9%. All RUs had a CDR comparable to or above the target value (5.5 per 1000 screenings). In 2013, the median RR had increased to 6.3%, and all RUs still had a performance outside the “target area”. Three RUs (RU4, RU10 and RU12) had a RR around or over 10% (Fig. 3B). After new target values were introduced in mid-2016 (Table 1), most RUs (except RU2, RU8 and RU9) continued to perform outside the “target area” (Fig. 3C). In the following 3 years, performance improved slightly. In 2019, five RUs (RU1, RU2, RU4, RU6 and RU7) performed on the border of the “target area” (Fig. 3D) and no RRs exceeded 10%.

### Variations and trends in RU performance, for subsequent screening

Similar to initial screening, the PPV-recall diagrams per year for 2010 to 2019 for subsequent screening show substantial variations in performance among the individual RUs. The lowest PPV observed during the study period was 21.2% (2014, RU12), based on a RR of 2.9% and a CDR of 6.0 per 1000 screenings. The highest PPV was 54.3% (2015, RU2) with a RR of 1.1% and a CDR of 6.0 per 1000 screenings. For overall performance across all units, the lowest median PPV was 31.1% (2013), and the highest median PPV was 37.2% (2019) (Table 3).

**Fig. 4:**
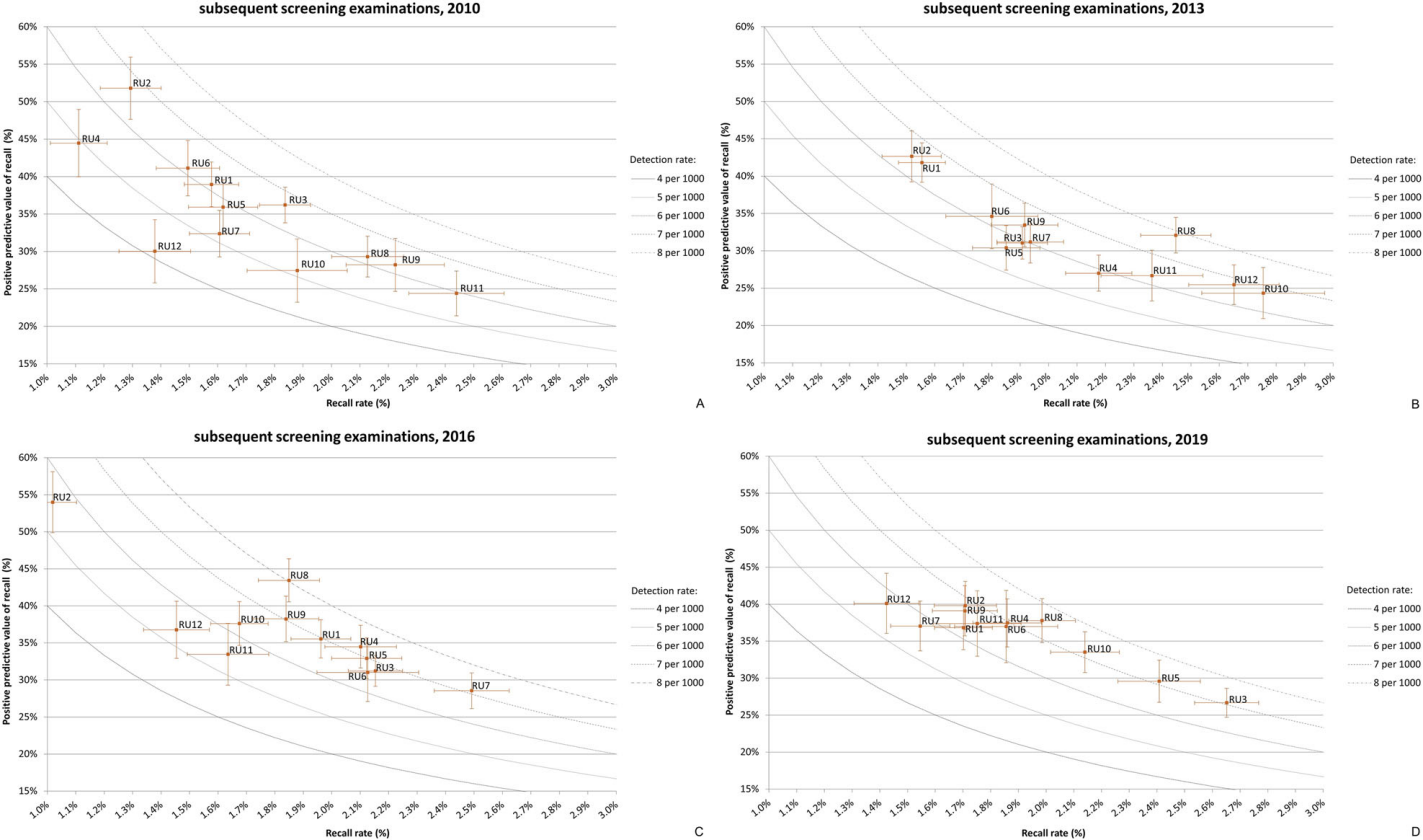
PPV-recall diagrams, with cancer detection rate (CDR) presented as isobars, for subsequent screening examinations for 2010 (**A**), 2013 (**B**), 2016 (**C**) and 2019 (**D**), showing how the 12 RUs performed relative to each other

Compared to initial screening, RUs more often met the target values. In 2010, 8 out of 12 RUs met all three target values (Fig. 4A). Of the four RUs outside the “target area”, three (RU8, RU9 and R11) had a RR above the target range of 1.3–2.0% and one (RU4) below. The median RR was 1.6% (Table 3).

Also similar to initial screening, the RR increased over the next 3 years. In 2013, the median RR was 1.9%, with five RUs (RU4, RU8, RU10-RU12) showing a RR above the target range (Fig. 4B). Between 2013 and 2016, overall performance increased. The median PPV increased from 31.1% to 35.0% (Table 3). New target values were introduced in mid-2016 (Table 1). In 2016, all RUs except for one (RU7) met all three target values (Fig. 4C). From 2016 to 2019, the overall performance increased slightly further. The median PPV increased to 37.2% in 2019 (Table 3). Now, two RUs (RU3 and RU5) performed outside the “target area” (Fig. 4D).

### Screening behaviour characteristics of the individual reading units

The PPV-recall diagrams per RU over all years provide insight into the screening behaviour characteristics of individual RUs. To illustrate this, we present two examples. We selected RU2 and RU12 because RU2 had the highest PPV observed during the study period for both initial (23.7%, 2014) and subsequent screening (54.3%, 2015), and RU12 had the lowest PPV for both initial (4.9%, 2014) and subsequent screening (21.2%, 2014).

**Fig. 5:**
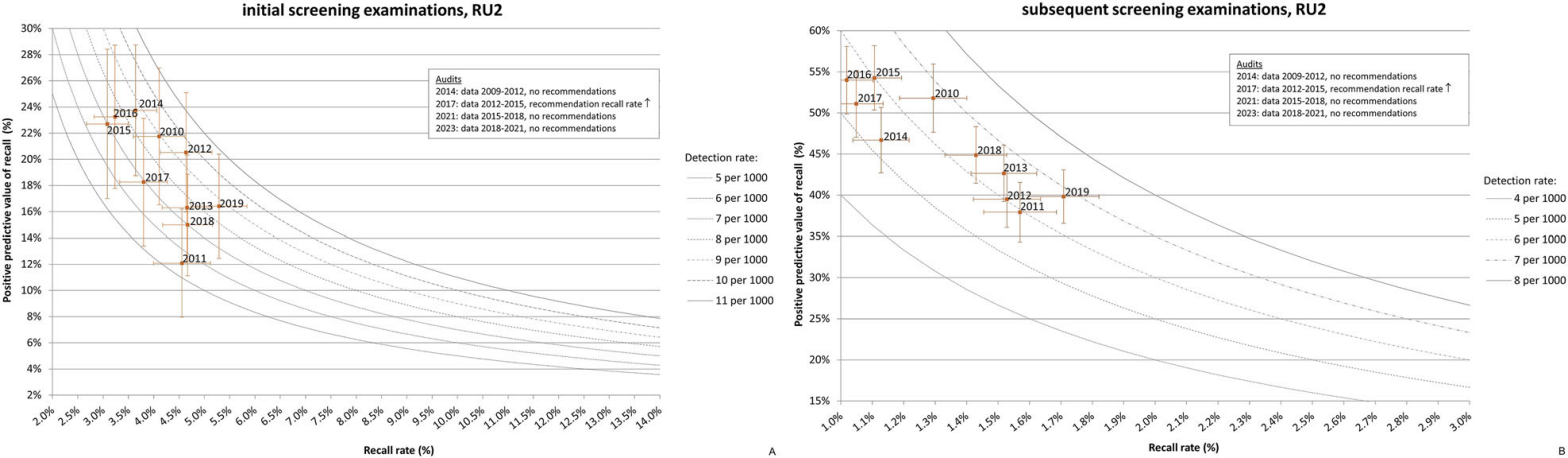
PPV-recall diagrams, with cancer detection rate (CDR) presented as isobars, for RU2 for initial (**A**) and subsequent screening examination (**B**), both over the period 2010–2019. The audit year, period of the audit data and audit recommendations are indicated in the frame

For RU2, the PPV-recall diagrams (Fig. 5) show a relatively low RR and high PPV. Four audits were conducted in the period 2010–2019. Based on the 2017 audit (data 2012–2015), RU2 was recommended to increase their RR, as the PPV-recall diagrams showed a high PPV alongside a declining RR for both initial and subsequent screening. Although the CDR met the target value, the audit team expected that the very low RR allowed for a slight increase, potentially resulting in an increased CDR. In the period after 2017, the RR increased while still meeting the target value, leading to a higher CDR in 2019.

**Fig. 6:**
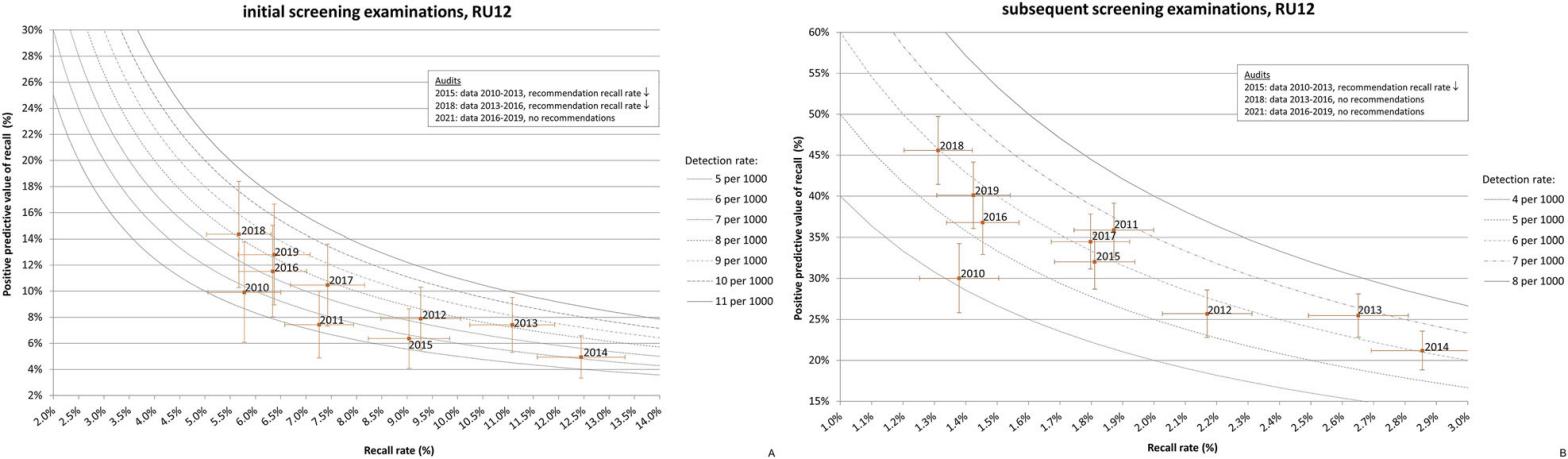
PPV-recall diagrams, with cancer detection rate (CDR) presented as isobars, for RU12 for initial (**A**) and subsequent screening examination (**B**), both over the period 2010–2019. The audit year, period of the audit data and audit recommendations are indicated in the frame

For RU12, the PPV-recall diagrams (Fig. 6) show that this RU is characterised by a very high RR and low PPV in the period 2012–2015. Three audits were conducted during the study period, with recommendations provided during the audits in 2015 and 2018. Based on the 2015 audit (data 2010–2013), the radiologists were strongly advised to reduce their RR, as their PPV was low and the RR had rapidly increased for both initial and subsequent screening. After 2015, the PPV-recall diagrams showed a considerable reduction in RR, meeting the target value for subsequent screening. However, for initial screening, the RR remained higher than the target value of 5.0%. During the 2018 audit (data 2013–2016), the radiologists were once again recommended to lower their RR, this time only for initial screening. In the period after 2018, the RR did not decrease.

### Audit recommendations and performance changes

A total of 42 audits were conducted across the 12 RUs, resulting in 37 recommendations: 24 for initial and 13 for subsequent screening. All recommendations concerned lowering, increasing or stabilising RR. The latter was regarding subsequent screening, combined with a recommendation to lower the RR only for initial screening.

For initial screening (Table 4), the RR changed in the desired direction for nine recommendations out of 24, with five RUs meeting the target value after 1 year. For six recommendations the RR remained unchanged or changed in the opposite direction, whereas for nine recommendations changes in RR could not be assessed (were outside study period).

**Table 4:** Summary of recommendations during audits conducted during the study period and changes in recall rate after the audit, regarding initial screening

Reading unit	Audit^a^	Evaluation period	Mean RR evaluation period^b^	Recommendation^c^	RR in the last year of evaluation period^d^	Difference in RR between the last year of the evaluation period and:			
						1st year after audit^e^	2nd year after audit^e^	3rd year after audit^e^	Target value is met after 1st year^f^
RU1	January 2019	2014–2017	5.9%	RR↓	6.5%	−2.1%	NA	NA	✓
RU2	April 2017	2012–2015	4.0%	RR↑	3.1%	+0.7%	+1.6%	+2.2%	✓
RU4	April 2015	2010–2013	7.5%	RR↓	9.8%	−1.4%	−0.5%	−1.6%	X
RU4	June 2018	2013–2016	9.0%	RR↓	9.3%	−4.1%	−3.9%	NA	✓
RU5	June 2015	2010–2013	5.9%	RR↓	7.2%	−0.2%	−0.3%	−1.0%	X
RU5	June 2018	2013–2016	6.6%	RR↓	6.9%	=0.0%	+1.2%	NA	X
RU6	March 2017	2012–2015	6.4%	RR↓	7.2%	−2.3%	−2.1%	−2.0%	✓
RU7	January 2016	2011–2014	4.9%	RR↓	6.0%	+1.8%	+3.1%	+1.4%	X
RU7	December 2018	2014–2017	7.4%	RR↓	9.1%	−3.9%	NA	NA	✓
RU10	December 2013	2010–2012	7.7%	RR↓	8.2%	−0.8%	−1.0%	−1.0%	X
RU10	November 2016	2012–2015	8.6%	RR↓	7.2%	+0.5%	+0.1%	+1.2%	X
RU11	June 2013	2010–2011	6.7%	RR↓	6.6%	+1.3%	−0.1%	+0.5%	X
RU11	May 2016	2011–2014	7.0%	RR↓	6.5%	+0.8%	−0.2%	−1.0%	X
RU12	February 2015	2010–2013	8.3%	RR↓	11.1%	−2.0%	−4.7%	−3.7%	X
RU12	March 2018	2013–2016	9.7%	RR↓	6.3%	−0.7%	=0.0%	NA	X

*RU* Reading unit, *RR* recall rate, *NA* not available

^a^ For nine audits, the changes in RR could not be assessed as the first year after the audit fell outside the study period (2020 or later), and are therefore not included in this table

^b^ At the time of the audit, all recommendations given by the audit team were based on the mean RR of the evaluation period

^c^ RR↓ = recommendation to decrease recall rate; RR↑ = recommendation to increase recall rate

^d^ For this study, the RR in the last year of the evaluation period was used as the ‘baseline’, against which changes in RR after the audit were compared

^e^ If the audit took place between 1 January and 30 June, the year in which the audit took place was also considered to be the first year after the audit; NA = not available, data outside study period

^f^ ✓ = the target value is met; X = the target value is not met

For subsequent screening (Table 5), the RR changed in the desired direction for eight recommendations, with six RUs meeting the target value after 1 year. For one RU, the RR increased while the recommendation was to stabilise, and for four RUs, changes in RR could not be assessed.

**Table 5:** Summary of recommendations during audits conducted during the study period and changes in recall rate after the audit, regarding subsequent screening

Reading unit	Audit^a^	Evaluation period	Mean RR evaluation period^b^	Recommendation^c^	RR in the last year of evaluation period^d^	Difference in RR between the last year of the evaluation period and:			
						1st year after audit^e^	2nd year after audit^e^	3rd year after audit^e^	Target value is met after 1st year^f^
RU2	April 2017	2012–2015	1.3%	RR↑	1.1%	−0.1%	+0.3%	+0.6%	X
RU5	June 2015	2010–2013	1.6%	RR=	1.9%	+0.1%	+0.3%	−0.1%	✓
RU5	June 2018	2013–2016	1.9%	RR=	2.1%	=0.0%	+0.3%	NA	✓
RU7	January 2016	2011–2014	1.8%	RR=	2.1%	+0.4%	+0.4%	−0.1%	X
RU7	December 2018	2014–2017	2.3%	RR↓	2.5%	−1.0%	NA	NA	✓
RU10	December 2013	2010–2012	2.1%	RR↓	2.5%	−0.4%	−0.7%	−0.8%	✓
RU11	June 2013	2010–2011	2.4%	RR↓	2.4%	−0.1%	−0.2%	−0.4%	X
RU11	May 2016	2011–2014	2.3%	RR↓	2.2%	−0.6%	−0.8%	−0.7%	✓
RU12	February 2015	2010–2013	2.0%	RR↓	2.7%	−0.8%	−1.2%	−0.9%	✓

*RU* Reading unit, *RR* recall rate, *NA* not available

^a^ For four audits, the changes in RR could not be assessed as the first year after the audit fell outside the study period (2020 or later), and are therefore not included in this table

^b^ At the time of the audit, all recommendations given by the audit team were based on the mean RR of the evaluation period

^c^ RR↓ = recommendation to decrease recall rate; RR↑ = recommendation to increase recall rate; RR= = recommendation to stabilise recall rate

^d^ For this study, the RR in the last year of the evaluation period was used as the ‘baseline’, against which changes in RR after the audit were compared

^e^ If the audit took place between 1 January and 30 June, the year in which the audit took place was also considered to be the first year after the audit; NA = not available, data outside study period

^f^ ✓ = the target value is met; X = the target value is not met

## Discussion

The PPV-recall diagrams demonstrated substantial variations in individual RU performance over a time period of 10 years, with PPVs ranging from 4.9 to 23.7% for initial and 21.2 to 54.3% for subsequent screening. Target values were less often met for initial (2010: 0 RUs; 2019: 5 RUs) than for subsequent screening (2010: 8 RUs; 2019: 10 RUs), resulting in more audit recommendations for initial screening (24 versus 13). All recommendations focused on adjusting RR, which often (9 out of 15 for initial and 8 out of 9 for subsequent screening) changed in the recommended direction, though not always sufficiently to meet target values (5 out of 9 for initial and 6 out of 8 for subsequent screening).

In 2001, Blanks et al from the UK described how they use the PPV-recall diagram as a tool to gain insight into the quality of screening programmes and how this can generate suggestions to improve screening quality [7]. Like our study, they found a substantial variation in reading performance between radiologist groups. However, they did not report whether providing recommendations to these screening groups resulted in improved screening outcomes.

Other papers related to the QA procedures of the UK breast cancer screening programme have also presented PPV-recall diagrams [13–15]. These studies highlighted the correlation between RR, CDR, and PPV and demonstrated that the PPV-recall diagram serves as a graphical tool to illustrate this relationship. However, similar to Blanks et al [7], these studies did not report recommendations to support quality improvement. A recent study on the Flemish breast cancer screening programme also uses the PPV-recall diagram for illustrative purposes only [16].

In addition, the study of Miglioretti et al [6] used the PPV-recall diagram to update performance criteria (target values) for screening radiologists. This study also focused on the interrelationship between RR, CDR, and PPV. Specifically, the authors suggested to define acceptable ranges for RR and PPV conditional on the CDR. In the PPV-recall diagram, these different zones were shaded using distinct colours. Based on our target values, we also defined an area in the PPV-recall diagram that indicated where all target values are met (see Fig. 1). Identifying the target area in the diagram visually demonstrates where screening outcomes deviate and specific recommendations should be issued.

It should be noted, though, that the target values specified for the Dutch breast cancer screening programme (see Table 1) differ considerably from those established in, e.g., the UK screening programme [7, 13–15] and the US [6]. Target values are inherently dependent on national policy, incidence of breast cancer, screening interval and age range of the screened population. Nonetheless, our approach within the QA programme could be generalised. The performance of a RU should meet the combination of established target values for RR, CDR, and PPV. If performance falls outside these thresholds, the RU receives a recommendation. The extent to which performance deviates from the target values determines the severity of the recommendation.

Our results suggest that the audit recommendations may not always be sufficient to ensure that RUs consistently meet the target values in subsequent years. There are several explanations for this finding. First, the 3-year audit frequency may be too infrequent. Several studies suggest that feedback should be based on recent performance to optimise effectiveness [17–21]. Second, feedback at the group level may be too indirect. The audit data are provided at the RU level. While optimal reading performance is a shared responsibility, improvement often depends on actions taken by individual radiologists. It is therefore plausible that the effectiveness of the audit process could increase if group performance feedback were combined with feedback on individual performance [20]. Third, the PPV-recall diagram may not be intuitive for radiologists and might require further explanation. Bowles and Geller concluded that radiologists generally prefer graphic displays over tables in audit feedback; however, their findings also revealed that radiologists found a graph similar to the PPV-recall diagram to be too complex [22]. Fourth, the recommendations lack clear guidance on how to achieve the goals. Our study showed that recommendations always focused on adjusting the RR, but did not suggest specific methods for achieving this. Feedback is likely to be more effective when accompanied by a plan with specific actions to reach targets [17, 20]. Radiologists might benefit from additional methods to optimise their recall decision, such as reviewing test sets with specific mammographic abnormalities, in order to identify which types of abnormalities were unnecessarily recalled in false-positive screenings and which false-negative screenings could have been detected in the previous screening round.

An alternative explanation why the provided feedback may not always lead to achieving the target values is that these target values might be unrealistic and require adjustment. In mid-2016, the target values for the RR were increased from 3.5% to 5.0% for initial screening and from 2.0% to 2.15% for subsequent screening. Still, RUs generally have too high a recall rate for initial screening. All target values were mainly based on the study of Otten et al [23], which was conducted with analogue screening examinations and in a laboratory setting, using test sets. This highlights the need for a new study based on digital mammography in daily screening practice to determine optimal target values for the RR. With this goal, the Recall and detection Of breast Cancer in Screening (ROCS) study was started within the Dutch breast cancer screening programme in 2019. Sechopoulos et al [24] published the study design in 2022, and the analysis for this study is ongoing.

Our study has certain limitations. First, we were unable to investigate whether there was a causal relationship between a recommendation and a change in performance after an audit, as it is not possible to separate the impact of an audit from other factors. Second, due to incomplete data regarding interval cancers during the audits, we could not compare the results of the PPV-recall diagrams with the sensitivity and specificity of the RUs.

In conclusion, our results show that the PPV-recall diagram is a suitable tool for monitoring the reading performance of groups of screening radiologists within a breast cancer screening programme. For the audit team, it provides insight into the interrelationship between RR, CDR, and PPV, and into performance variations, which can help in formulating recommendations for improvement. However, for individual radiologists, audit feedback based on the PPV-recall diagram on the group level alone may not always be sufficient to achieve these improvements. To better support radiologists, feedback on overall group performance could be combined with feedback on individual performance, specific action plans for improvement could be provided, and feedback could be presented in a clearer and more intuitive way.

## Supplementary information

ELECTRONIC SUPPLEMENTARY MATERIAL

## Abbreviations

CDR: Cancer detection rate
IQR: Interquartile range
LRCB: Dutch Expert Centre for Screening
PPV: Positive predictive value of recall
QA: Quality assurance
RR: Recall rate
RUs: Reading units (groups of radiologists)

## References

[1] Dibden A, Offman J, Duffy SW, Gabe R (2020) Worldwide review and meta-analysis of cohort studies measuring the effect of mammography screening programmes on incidence-based breast cancer mortality. Cancers (Basel) 12:976 32326646 10.3390/cancers12040976 PMC7226343

[2] Marmot MG, Altman DG, Cameron DA, Dewar JA, Thompson SG, Wilcox M (2013) The benefits and harms of breast cancer screening: an independent review. Br J Cancer 108:2205–2240 23744281 10.1038/bjc.2013.177 PMC3693450

[3] Perry N, Broeders M, DeWolf C, Törnberg S, Holland R, Von Karsa L (2006) European guidelines for quality assurance in breast cancer screening and diagnosis, 4th edn. Office for Official Publications of the European Communities, Luxembourg 10.1093/annonc/mdm481 18024988

[4] European Commission: Joint Research Centre (2025) European quality assurance scheme for breast cancer services. Publications Office of the European Union, Luxembourg. Available via https://data.europa.eu/doi/10.2760/7067385. Accessed 14 Apr 2025

[5] U.S. Food and Drug Administration (2023) Mammography quality standards. Available via https://www.fda.gov/radiation-emitting-products/mammography-quality-standards-act-and-program. Accessed 26 Nov 2024

[6] Miglioretti DL, Ichikaw L, Smith RA et al (2015) Criteria for identifying radiologists with acceptable screening mammography interpretive performance on basis of multiple performance measures. AJR Am J Roentgenol 204:W486–W491 25794100 10.2214/AJR.13.12313 PMC4369798

[7] Blanks RG, Moss SM, Wallis MG (2001) Monitoring and evaluating the UK National Health Service Breast Screening Programme: evaluating the variation in radiological performance between individual programmes using PPV-referral diagrams. J Med Screen 8:24–28 11373846 10.1136/jms.8.1.24

[8] Geertse TD, Holland R, Timmers JM et al (2015) Value of audits in breast cancer screening quality assurance programmes. Eur Radiol 25:3338–3347 25903711 10.1007/s00330-015-3744-x

[9] Dutch Expert Centre for Screening (2022) Audit protocol breast cancer screening. Available via https://lrcb.nl/wp-content/uploads/2022/04/Visitatieprotocol-borstkankerscreening-versie-2022.pdf. Accessed 26 Nov 2024

[10] IKNL (2018/2019) National evaluation of breast cancer screening in the Netherlands. Available via https://iknl.nl/getmedia/cc1b7da5-28aa-43e6-b70a-b3dadf08ab89/monitor-bevolkingsonderzoek-borstkanker-2018-2019.pdf. Accessed 26 Nov 2024

[11] Bluekens AM, Holland R, Karssemeijer N, Broeders MJ, den Heeten GJ (2012) Comparison of digital screening mammography and screen-film mammography in the early detection of clinically relevant cancers: a multicenter study. Radiology 265:707–714 23033499 10.1148/radiol.12111461

[12] Dutch Expert Centre for Screening (2015) Advies en voorstel voor streefwaarde m.b.t. hoogte verwijscijfer [Advice and proposal for target value with regard to the level of the recall rate]. Dutch Expert Centre for Screening, Netherlands

[13] Perry NM (2003) Interpretive skills in the National Health Service Breast Screening Programme: performance indicators and remedial measures. Semin Breast Dis 6:108–113

[14] Bennett RL, Blanks RG (2007) Should a standard be defined for the positive predictive value (PPV) of recall in the UK NHS Breast Screening Programme? Breast 16:55–59 16904891 10.1016/j.breast.2006.05.008

[15] Cohen SL, Blanks RG, Jenkins J, Kearins O (2018) Role of performance metrics in breast screening imaging—where are we and where should we be? Clin Radiol 73:381–388 29395223 10.1016/j.crad.2017.12.012

[16] Goossens M, De Brabander I, De Grève J et al (2019) Flemish breast cancer screening programme: 15 years of key performance indicators (2002–2016). BMC Cancer 19:1012 31660890 10.1186/s12885-019-6230-z PMC6819643

[17] Ivers N, Jamtvedt G, Flottorp S et al (2012) Audit and feedback: effects on professional practice and healthcare outcomes. Cochrane Database Syst Rev 2012:CD000259 10.1002/14651858.CD000259.pub3 PMC11338587 22696318

[18] Ivers N, Yogasingam S, Lacroix M et al (2025) Audit and feedback: effects on professional practice. Cochrane Database Syst Rev 2025:CD000259 10.1002/14651858.CD000259.pub4 PMC11934852 40130784

[19] Brehaut JC, Colquhoun HL, Eva KW et al (2016) Practice feedback interventions: 15 suggestions for optimizing effectiveness. Ann Intern Med 164:435–441 26903136 10.7326/M15-2248

[20] Brown B, Gude WT, Blakeman T et al (2019) Clinical performance feedback intervention theory (CP-FIT): a new theory for designing, implementing, and evaluating feedback in health care based on a systematic review and meta-synthesis of qualitative research. Implement Sci 14:1–25 31027495 10.1186/s13012-019-0883-5 PMC6486695

[21] Hofvind S, Bennett RL, Brisson J et al (2016) Audit feedback on reading performance of screening mammograms: an international comparison. J Med Screen 23:150–159 26892191 10.1177/0969141315610790

[22] Bowles EJA, Geller BM (2009) Best ways to provide feedback to radiologists on mammography performance. AJR Am J Roentgenol 193:157–164 19542408 10.2214/AJR.08.2051 PMC2714544

[23] Otten JD, Karssemeijer N, Hendriks JH et al (2005) Effect of recall rate on earlier screen detection of breast cancers based on the Dutch performance indicators. J Natl Cancer Inst 97:748–754 15900044 10.1093/jnci/dji131

[24] Sechopoulos I, Abbey CK, van der Waal D et al (2022) Evaluation of reader performance during interpretation of breast cancer screening: the Recall and detection Of breast Cancer in Screening (ROCS) trial study design. Eur Radiol 32:7463–7469 35482123 10.1007/s00330-022-08820-5 PMC9668759

