# Supplementary material for: Applying the “positive predictive value–recall diagram” to monitor performance and provide recommendations for screening radiologists
Source: Eur Radiol. 2025 Sep 4;36(3):1919–31. doi: 10.1007/s00330-025-11978-3 (PMC12963250; doi:10.1007/s00330-025-11978-3)
Supplement: Supplementary file 1 — ELECTRONIC SUPPLEMENTARY MATERIAL [file 330_2025_11978_MOESM1_ESM.pdf]

Applying the "positive predictive value—recall diagram" to monitor performance and provide recommendations for screening radiologists

ELECTRONIC SUPPLEMENTARY MATERIAL

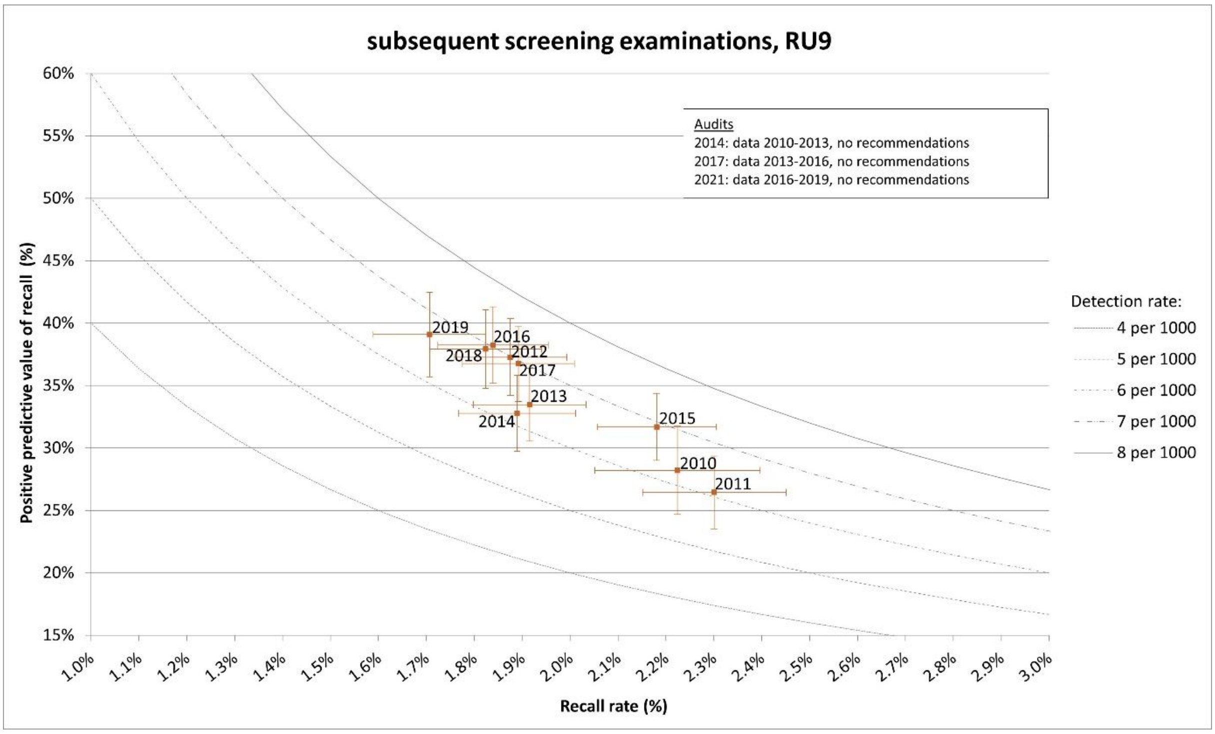

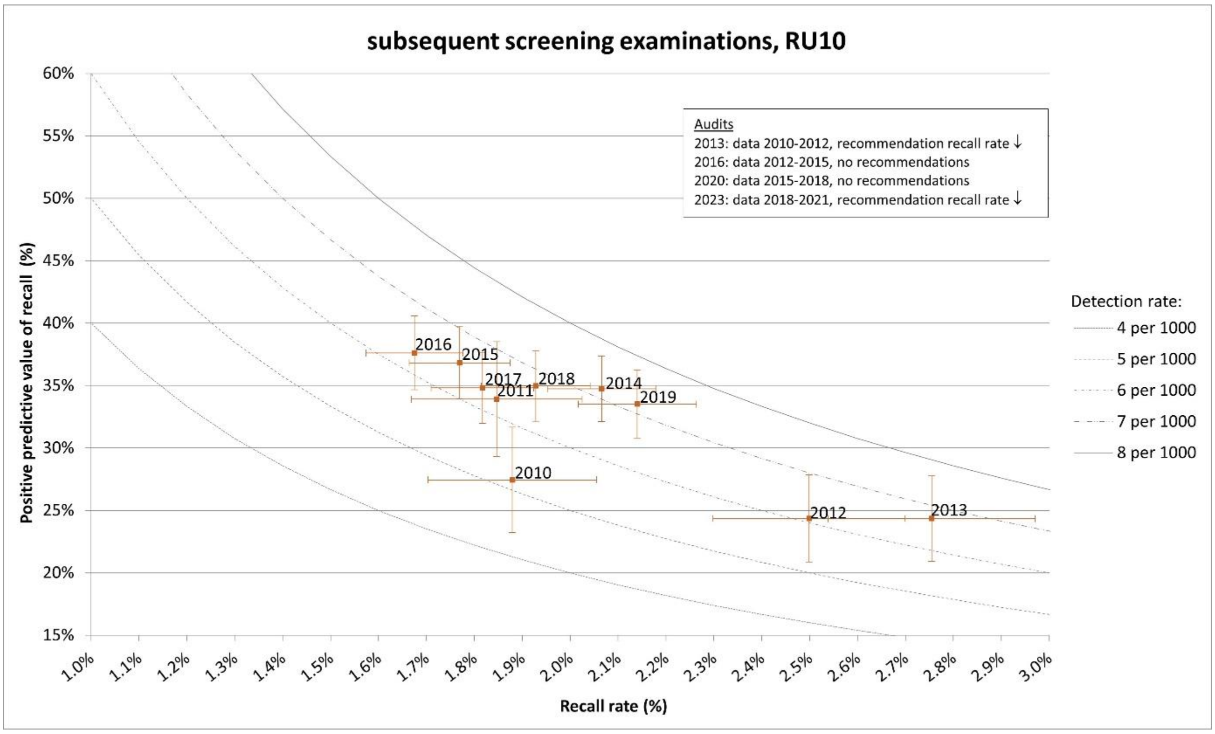

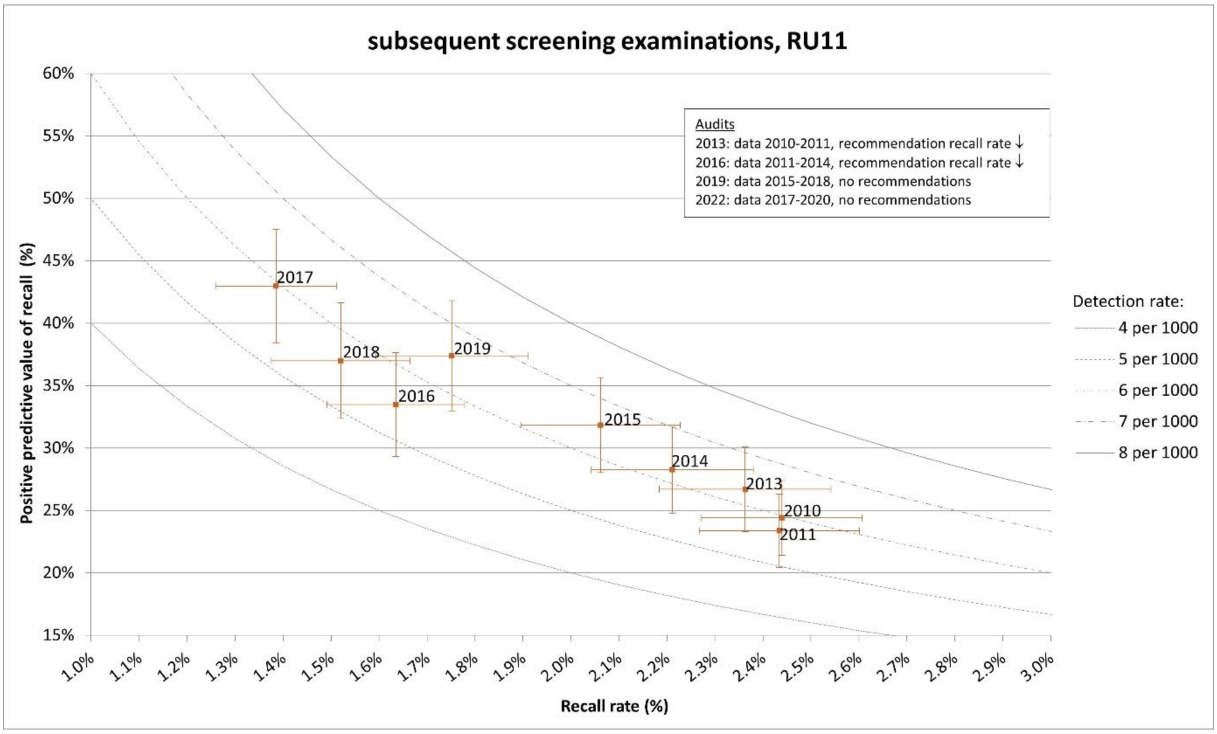
subsequent screening examinations, RU11
Audits
2013: data 2010-2011, recommendation recall rate ↓
2016: data 2011-2014, recommendation recall rate ↓
2019: data 2015-2018, no recommendations
2022: data 2017-2020, no recommendations
Detection rate:
4 per 1000
5 per 1000
6 per 1000
7 per 1000
8 per 1000
Positive predictive value of recall (%)
Recall rate (%)
2017
2018
2019
2016
2015
2014
2013
2010
2011

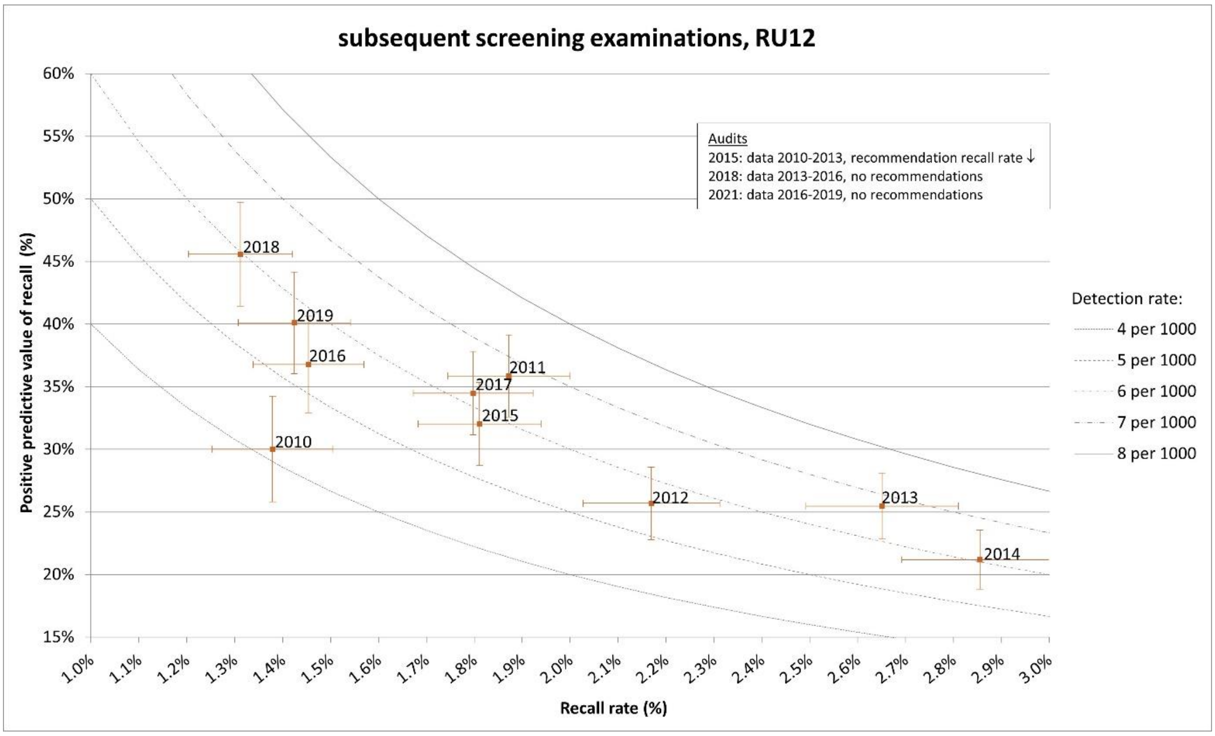
subsequent screening examinations, RU12
Audits
2015: data 2010-2013, recommendation recall rate ↓
2018: data 2013-2016, no recommendations
2021: data 2016-2019, no recommendations
Detection rate:
4 per 1000
5 per 1000
6 per 1000
7 per 1000
8 per 1000
Positive predictive value of recall (%)
Recall rate (%)
2018
2019
2016
2010
2011
2017
2015
2012
2013
2014

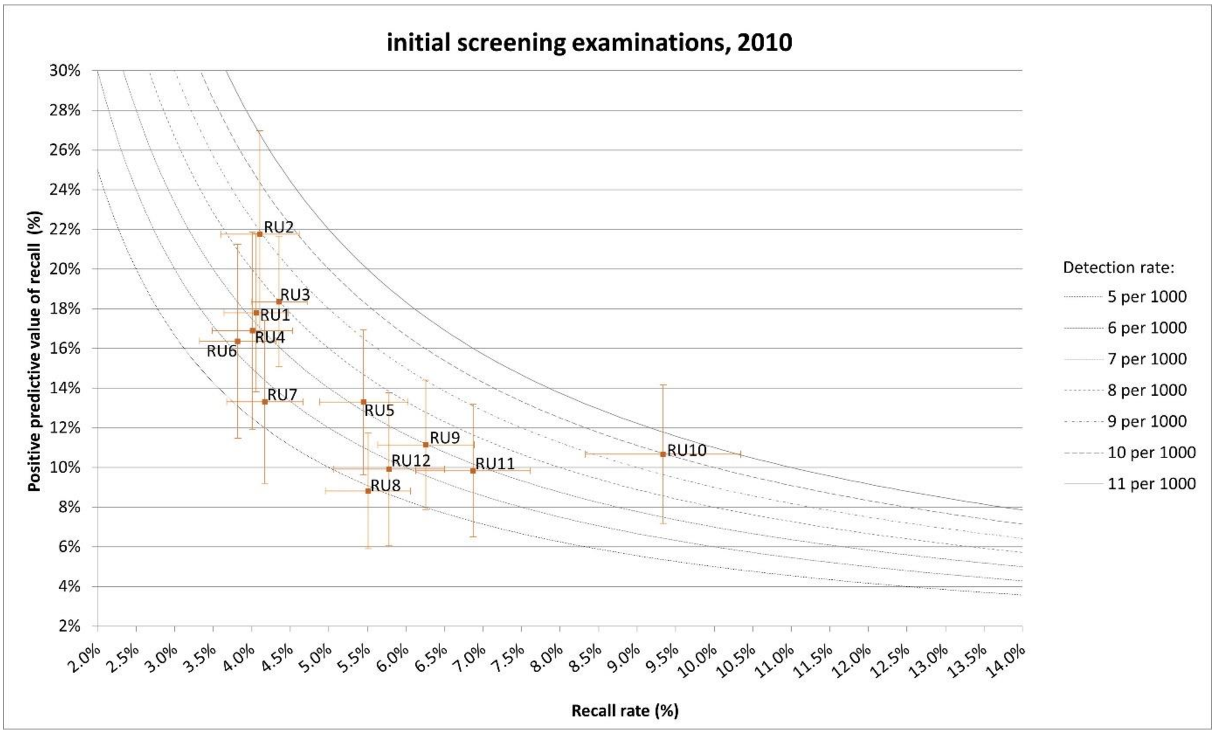
initial screening examinations, 2010
Positive predictive value of recall (%)
Recall rate (%)
RU1
RU2
RU3
RU4
RU5
RU6
RU7
RU8
RU9
RU10
RU11
RU12
Detection rate:
5 per 1000
6 per 1000
7 per 1000
8 per 1000
9 per 1000
10 per 1000
11 per 1000

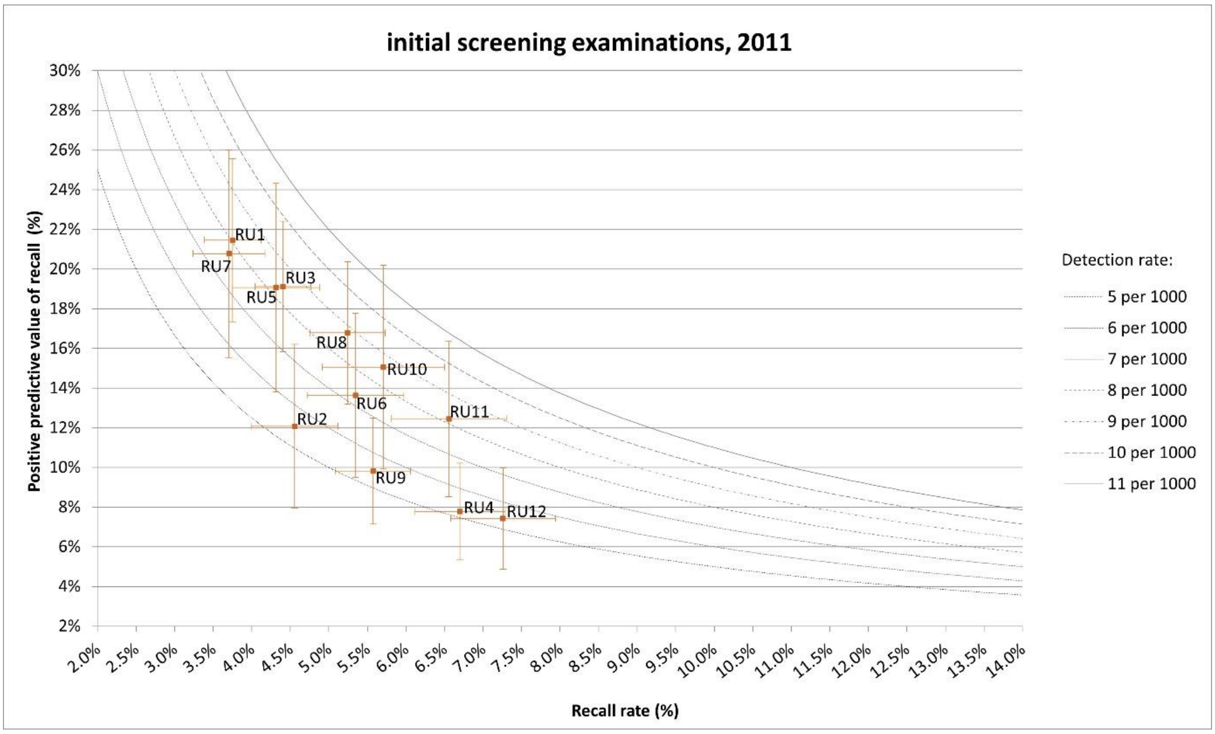
initial screening examinations, 2011
Positive predictive value of recall (%)
Recall rate (%)
RU1
RU7
RU3
RU5
RU8
RU10
RU6
RU11
RU2
RU9
RU4
RU12
Detection rate:
5 per 1000
6 per 1000
7 per 1000
8 per 1000
9 per 1000
10 per 1000
11 per 1000

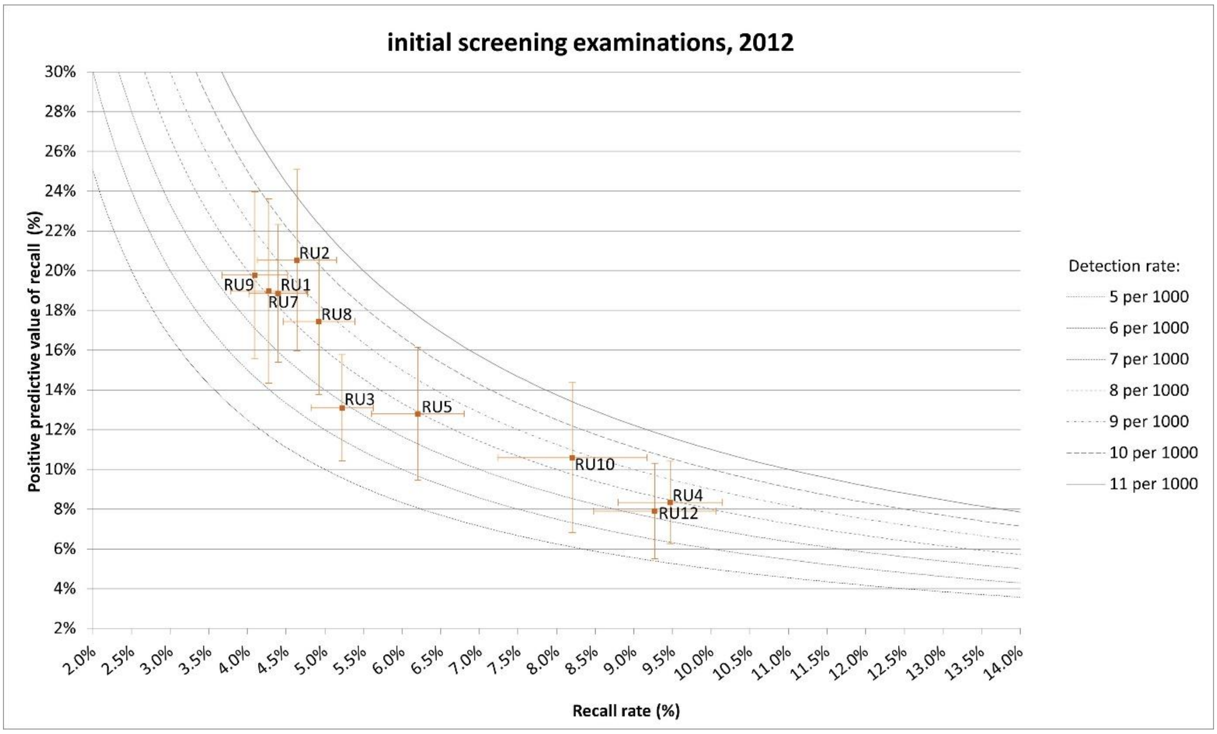
initial screening examinations, 2012
Positive predictive value of recall (%)
Recall rate (%)
Detection rate:
5 per 1000
6 per 1000
7 per 1000
8 per 1000
9 per 1000
10 per 1000
11 per 1000
RU1
RU2
RU3
RU4
RU5
RU7
RU8
RU9
RU10
RU12

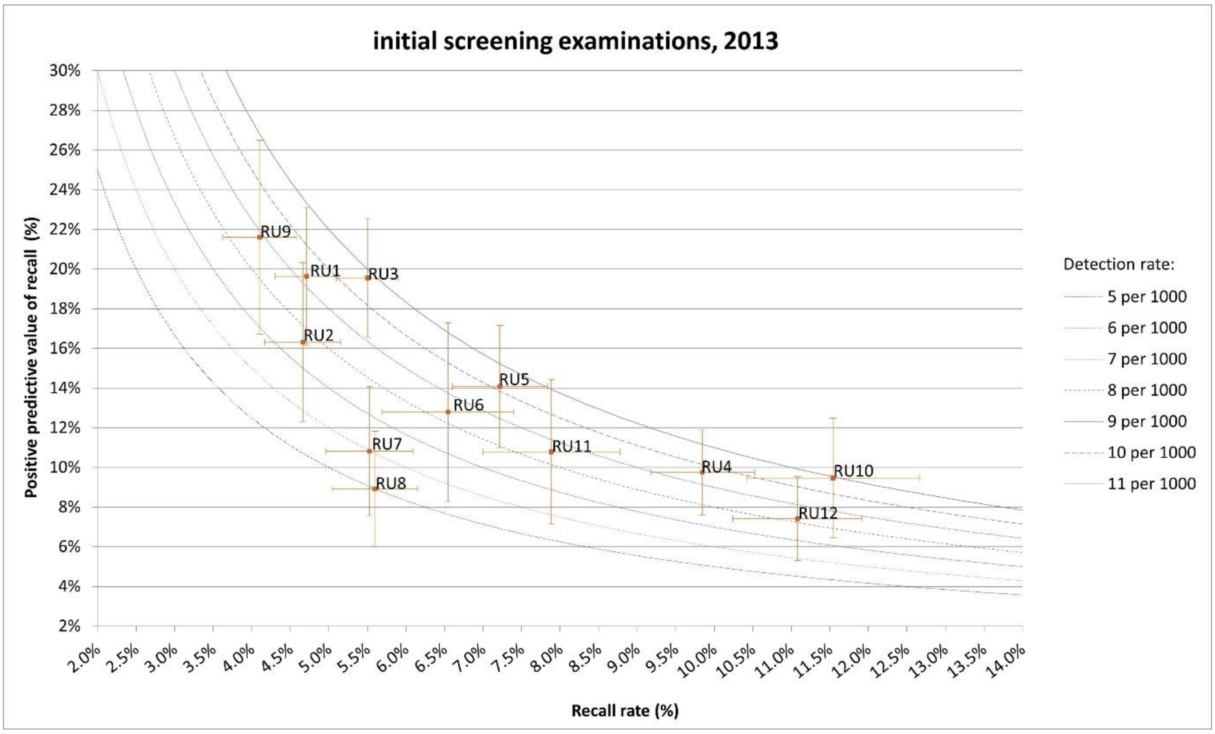
initial screening examinations, 2013
Positive predictive value of recall (%)
Recall rate (%)
Detection rate:
5 per 1000
6 per 1000
7 per 1000
8 per 1000
9 per 1000
10 per 1000
11 per 1000
RU1
RU2
RU3
RU4
RU5
RU6
RU7
RU8
RU9
RU10
RU11
RU12

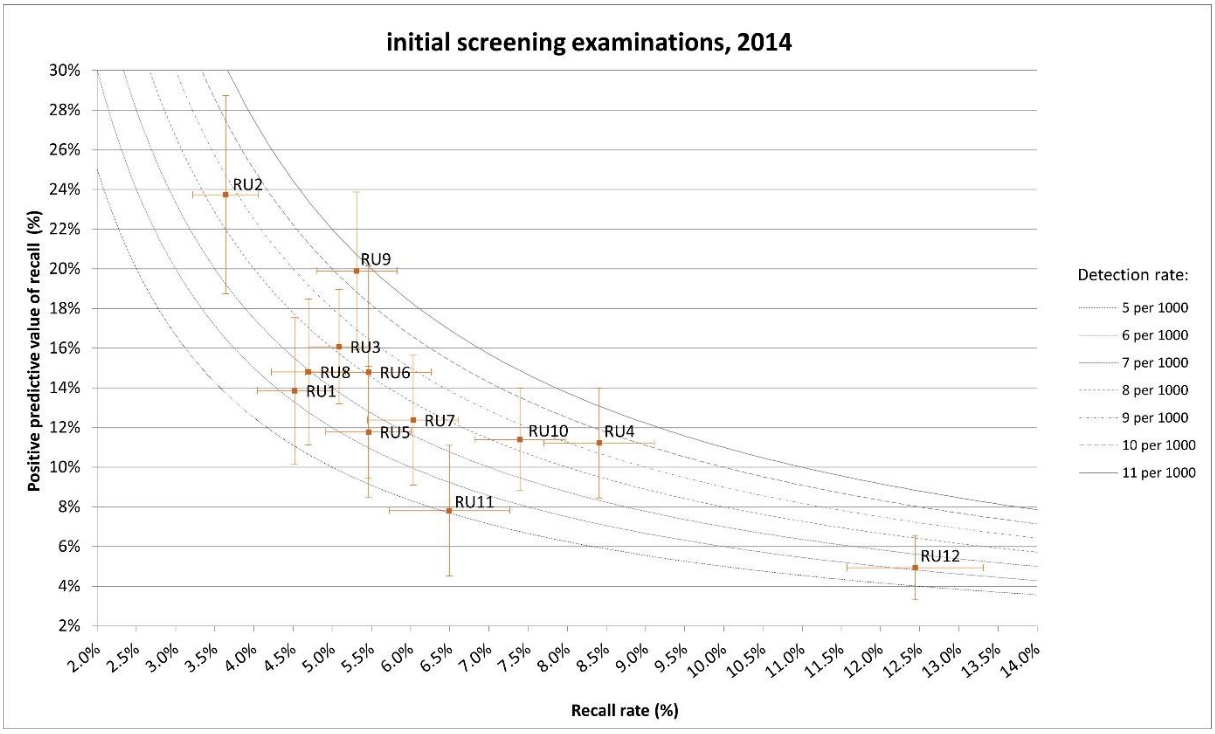

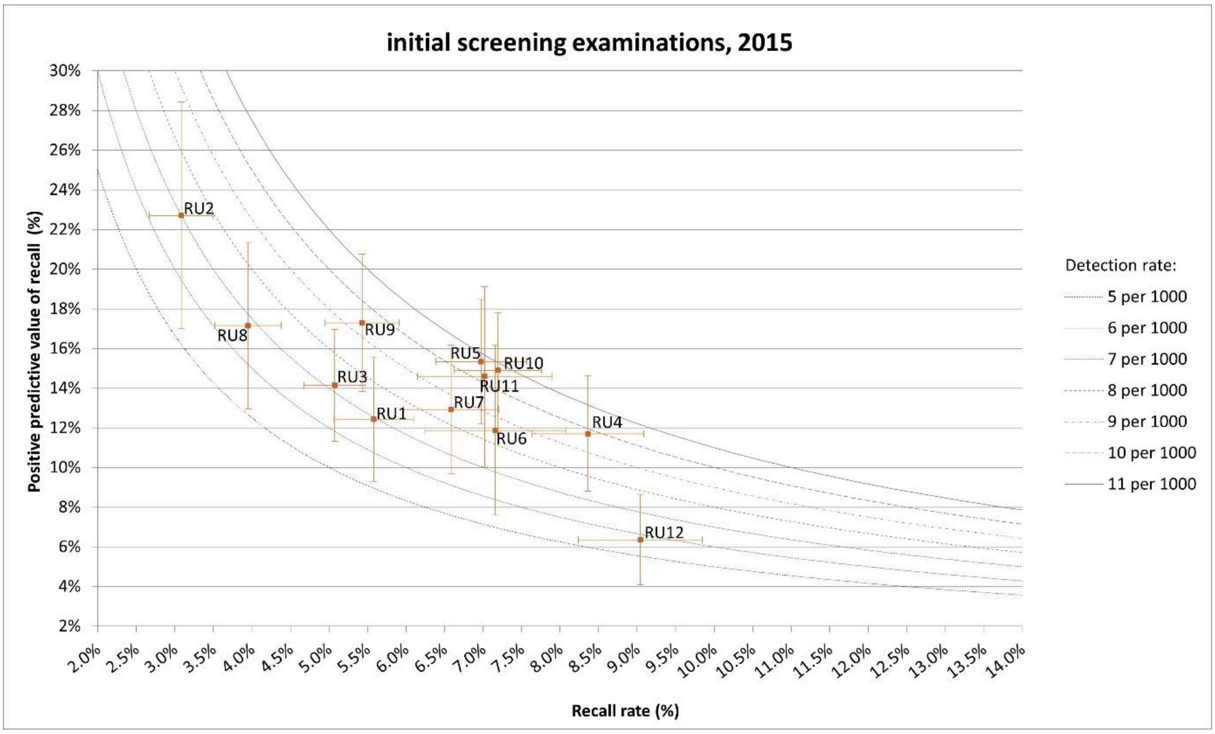

Eur Radiol (2025) Geertse TD, Tetteroo E, Smid-Geirnaerdt MJA, et al

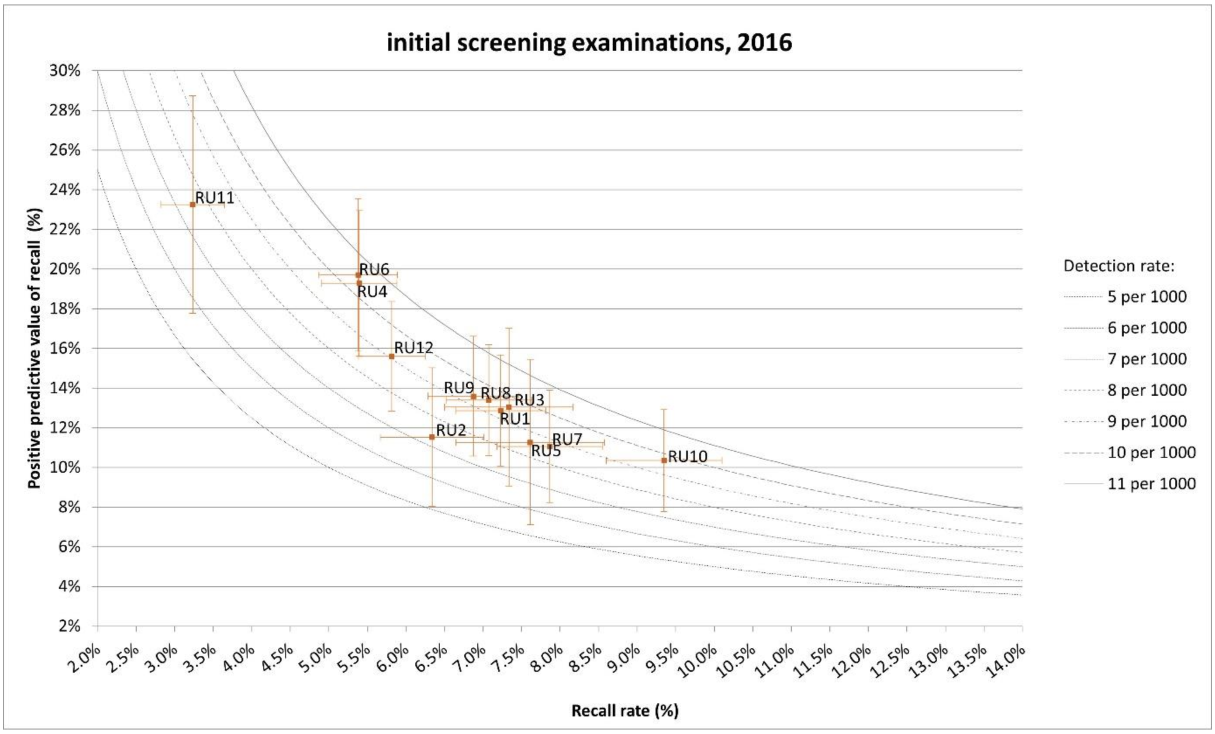
initial screening examinations, 2016
Positive predictive value of recall (%)
Recall rate (%)
Detection rate:
5 per 1000
6 per 1000
7 per 1000
8 per 1000
9 per 1000
10 per 1000
11 per 1000
RU11
RU6
RU4
RU12
RU9
RU8
RU3
RU1
RU2
RU7
RU5
RU10

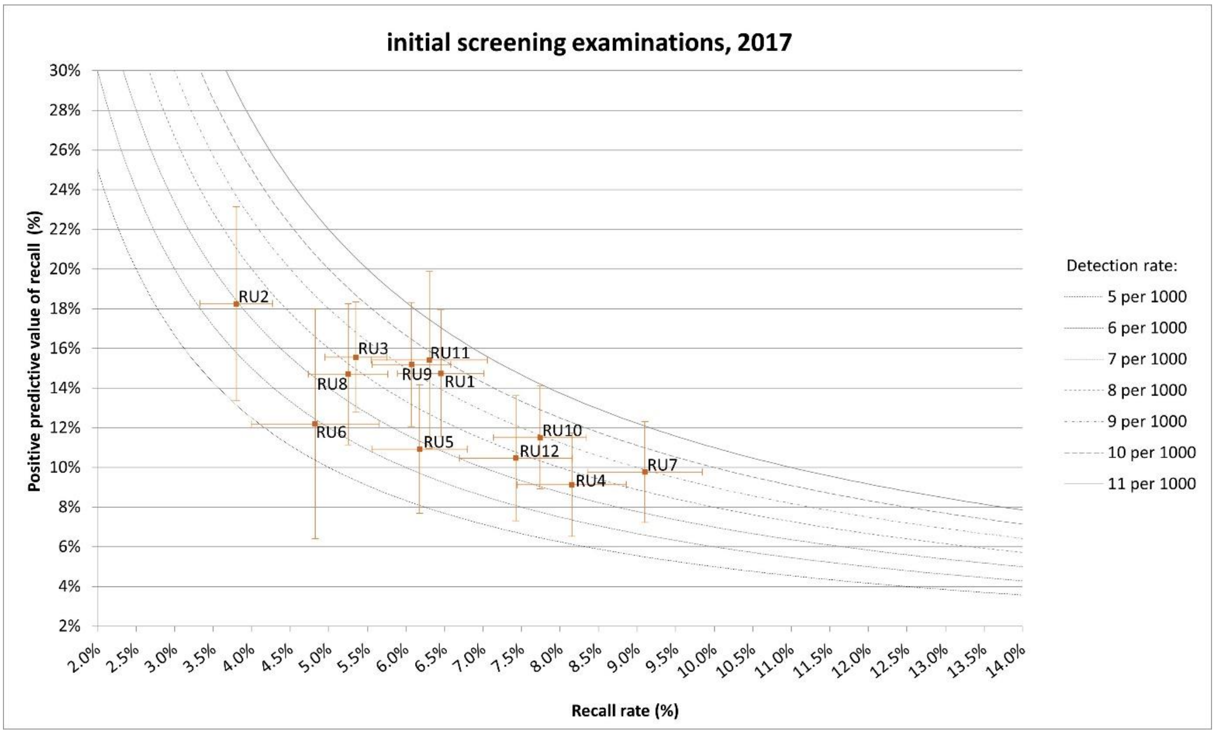
initial screening examinations, 2017
Positive predictive value of recall (%)
Recall rate (%)
Detection rate:
5 per 1000
6 per 1000
7 per 1000
8 per 1000
9 per 1000
10 per 1000
11 per 1000
RU2
RU3
RU11
RU8
RU9
RU1
RU6
RU5
RU10
RU12
RU7
RU4

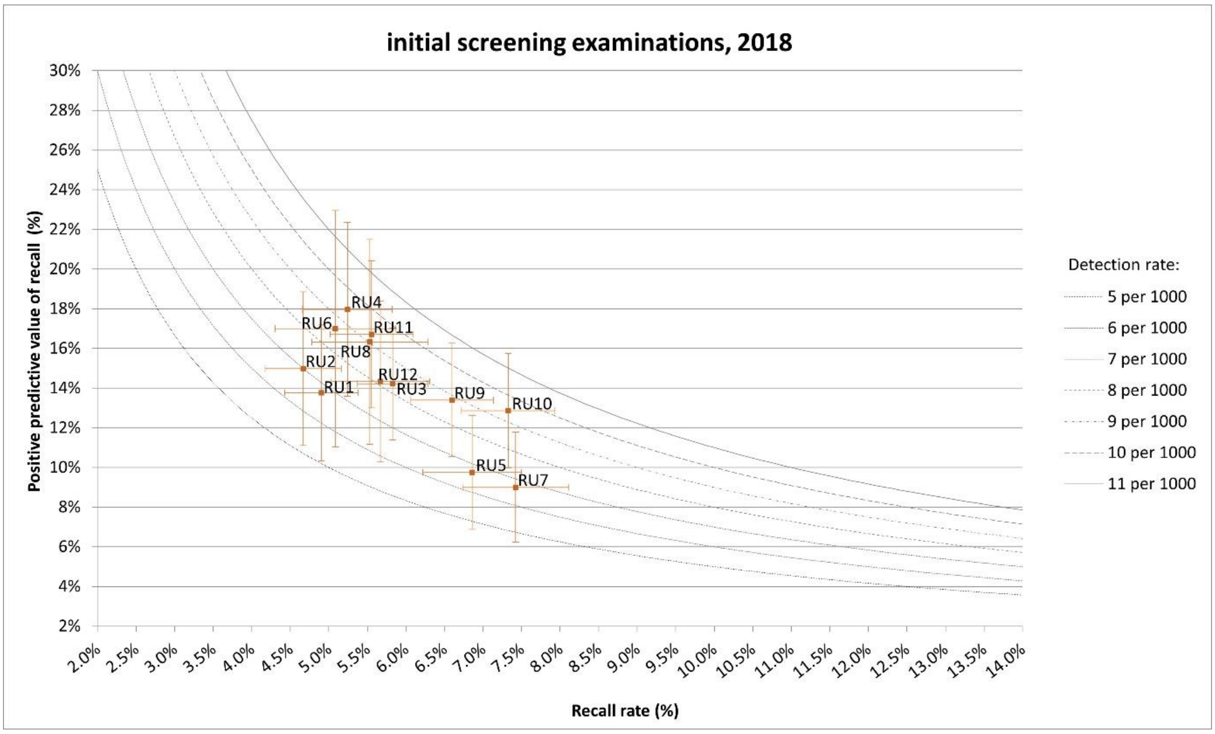
initial screening examinations, 2018
Positive predictive value of recall (%)
Recall rate (%)
Detection rate:
5 per 1000
6 per 1000
7 per 1000
8 per 1000
9 per 1000
10 per 1000
11 per 1000
RU1
RU2
RU3
RU4
RU5
RU6
RU7
RU8
RU9
RU10
RU11
RU12

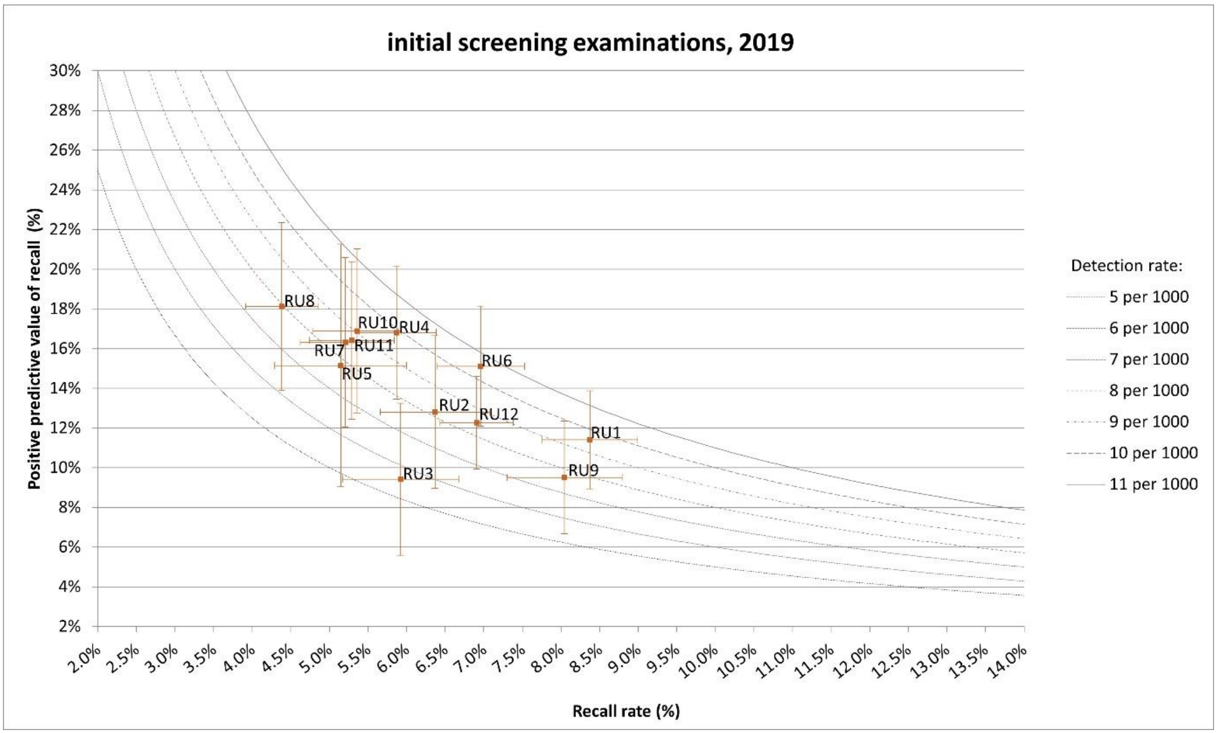
initial screening examinations, 2019
Positive predictive value of recall (%)
Recall rate (%)
Detection rate:
5 per 1000
6 per 1000
7 per 1000
8 per 1000
9 per 1000
10 per 1000
11 per 1000
RU1
RU2
RU3
RU4
RU5
RU6
RU7
RU8
RU9
RU10
RU11
RU12

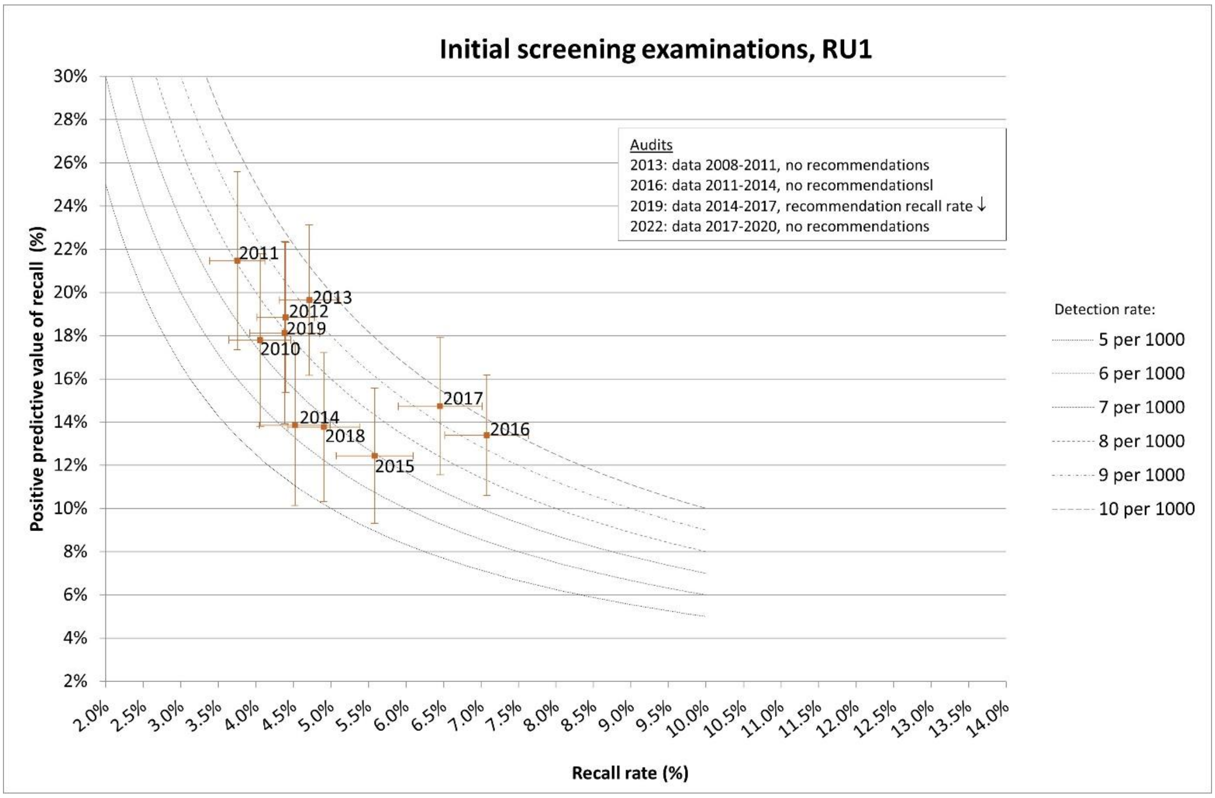
Initial screening examinations, RU1
Audits
2013: data 2008-2011, no recommendations
2016: data 2011-2014, no recommendationsl
2019: data 2014-2017, recommendation recall rate ↓
2022: data 2017-2020, no recommendations
Detection rate:
5 per 1000
6 per 1000
7 per 1000
8 per 1000
9 per 1000
10 per 1000
Positive predictive value of recall (%)
Recall rate (%)

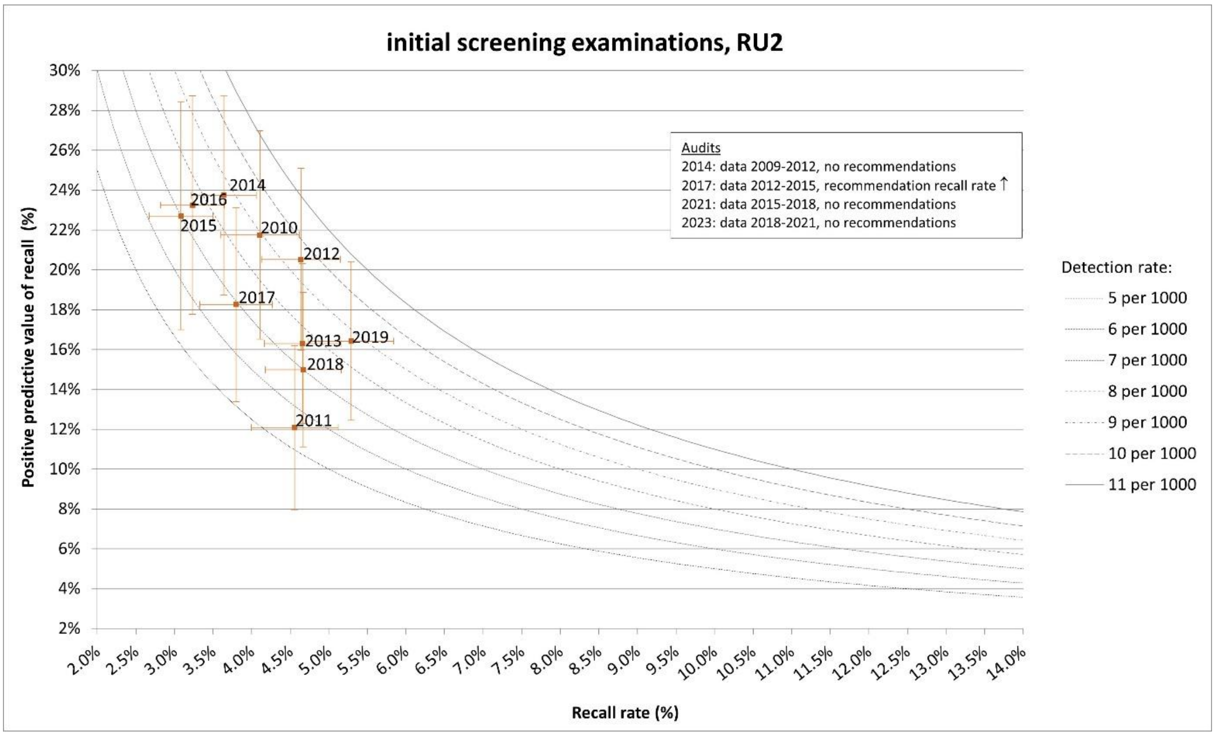
initial screening examinations, RU2
Audits
2014: data 2009-2012, no recommendations
2017: data 2012-2015, recommendation recall rate ↑
2021: data 2015-2018, no recommendations
2023: data 2018-2021, no recommendations
Detection rate:
5 per 1000
6 per 1000
7 per 1000
8 per 1000
9 per 1000
10 per 1000
11 per 1000
Positive predictive value of recall (%)
Recall rate (%)

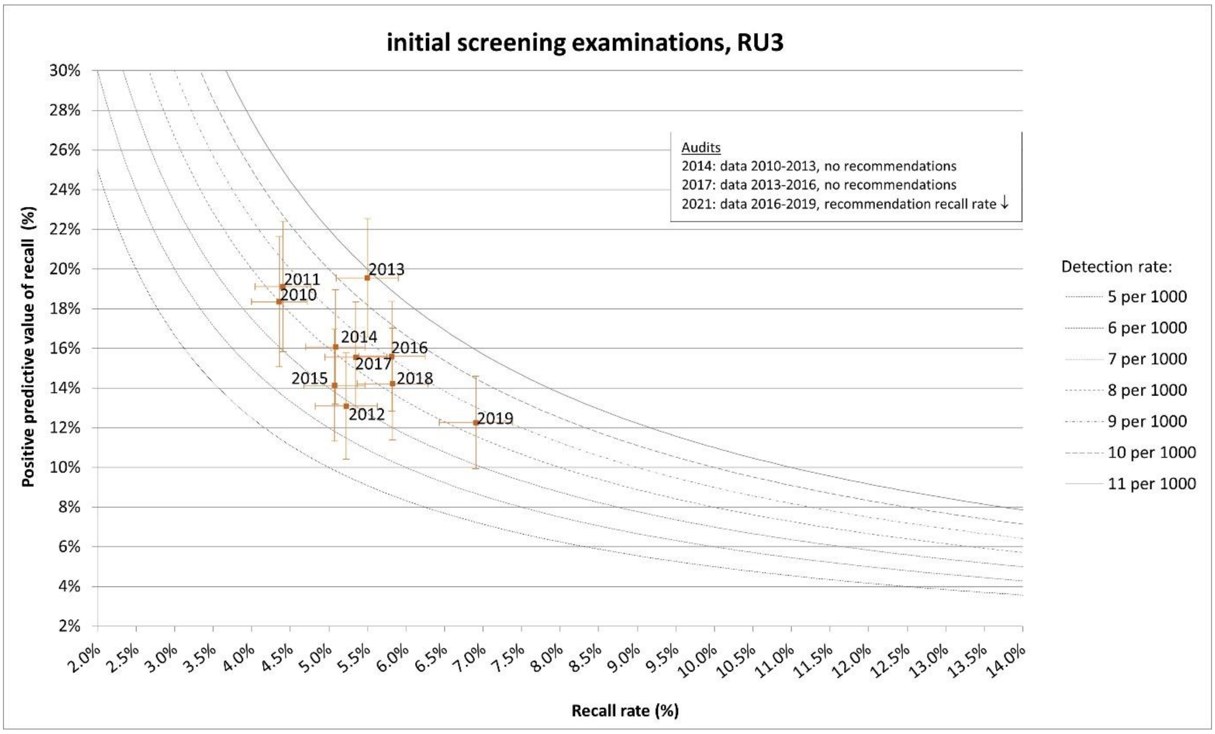
initial screening examinations, RU3
Audits
2014: data 2010-2013, no recommendations
2017: data 2013-2016, no recommendations
2021: data 2016-2019, recommendation recall rate ↓
Detection rate:
5 per 1000
6 per 1000
7 per 1000
8 per 1000
9 per 1000
10 per 1000
11 per 1000
Positive predictive value of recall (%)
Recall rate (%)
2010
2011
2012
2013
2014
2015
2016
2017
2018
2019

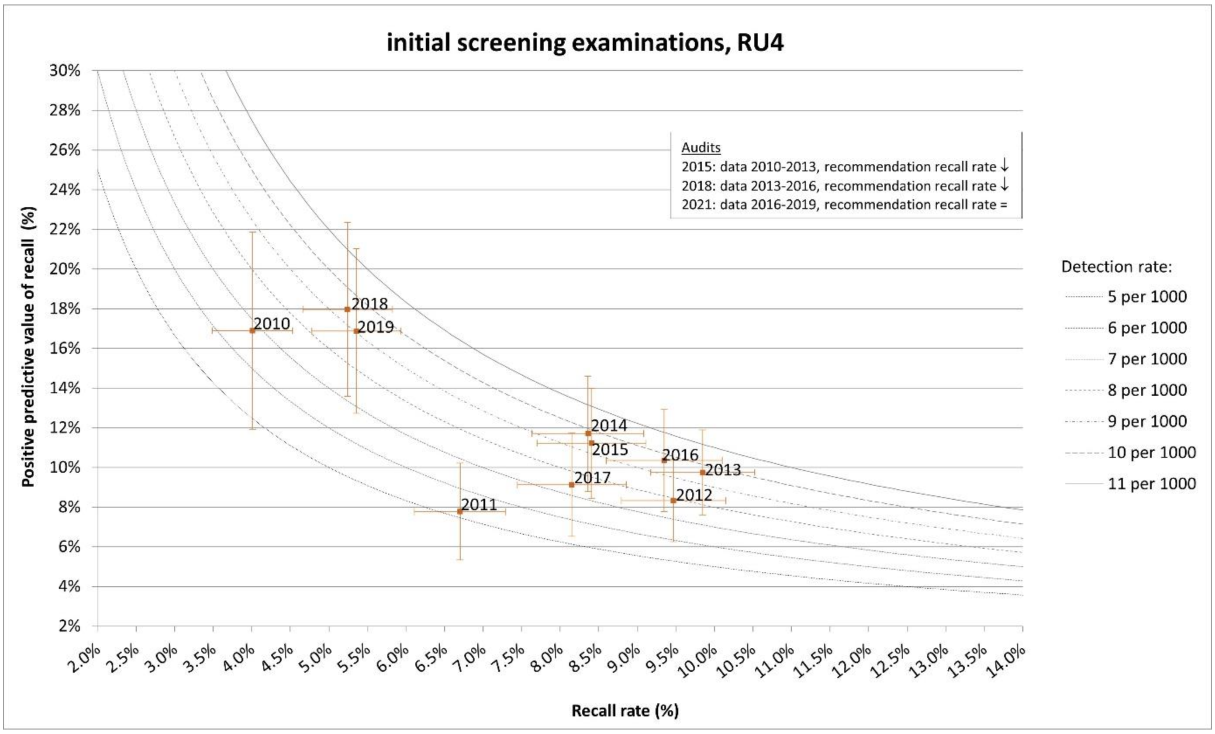
initial screening examinations, RU4
Audits
2015: data 2010-2013, recommendation recall rate ↓
2018: data 2013-2016, recommendation recall rate ↓
2021: data 2016-2019, recommendation recall rate =
Detection rate:
5 per 1000
6 per 1000
7 per 1000
8 per 1000
9 per 1000
10 per 1000
11 per 1000
Positive predictive value of recall (%)
Recall rate (%)
2010
2011
2012
2013
2014
2015
2016
2017
2018
2019

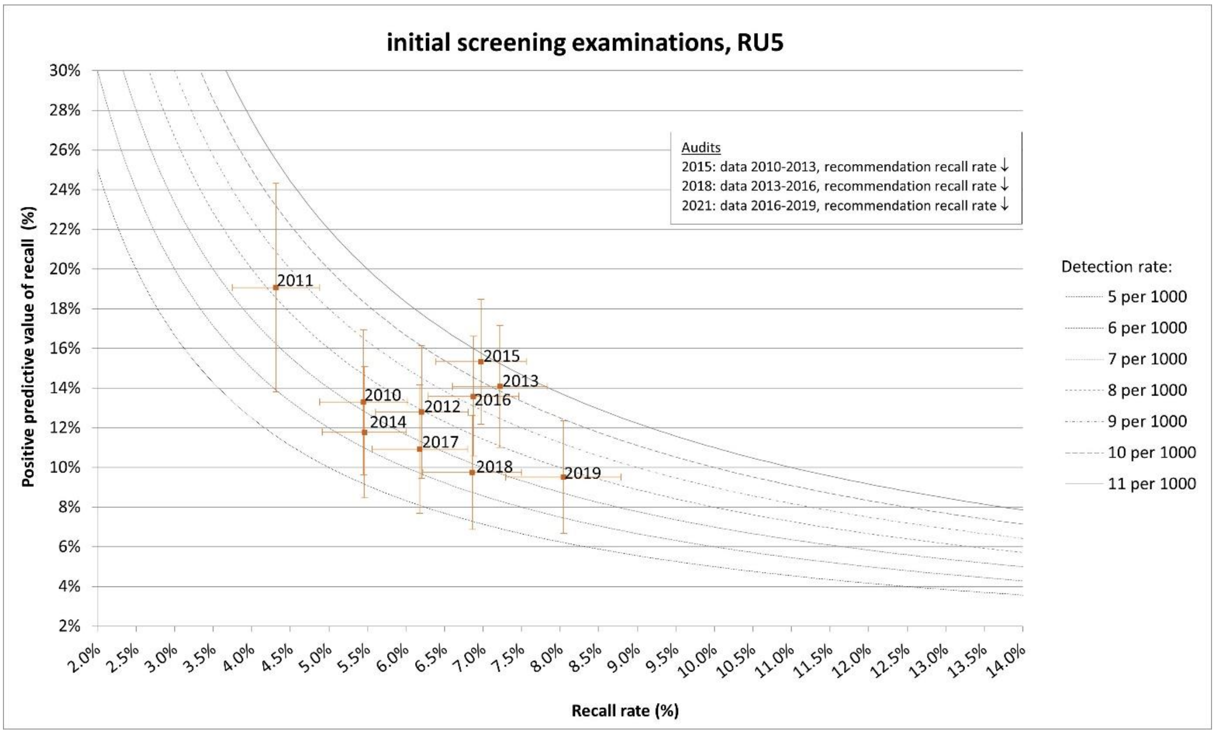

initial screening examinations, RU5
Audits
2015: data 2010-2013, recommendation recall rate ↓
2018: data 2013-2016, recommendation recall rate ↓
2021: data 2016-2019, recommendation recall rate ↓
Detection rate:
5 per 1000
6 per 1000
7 per 1000
8 per 1000
9 per 1000
10 per 1000
11 per 1000
Positive predictive value of recall (%)
Recall rate (%)

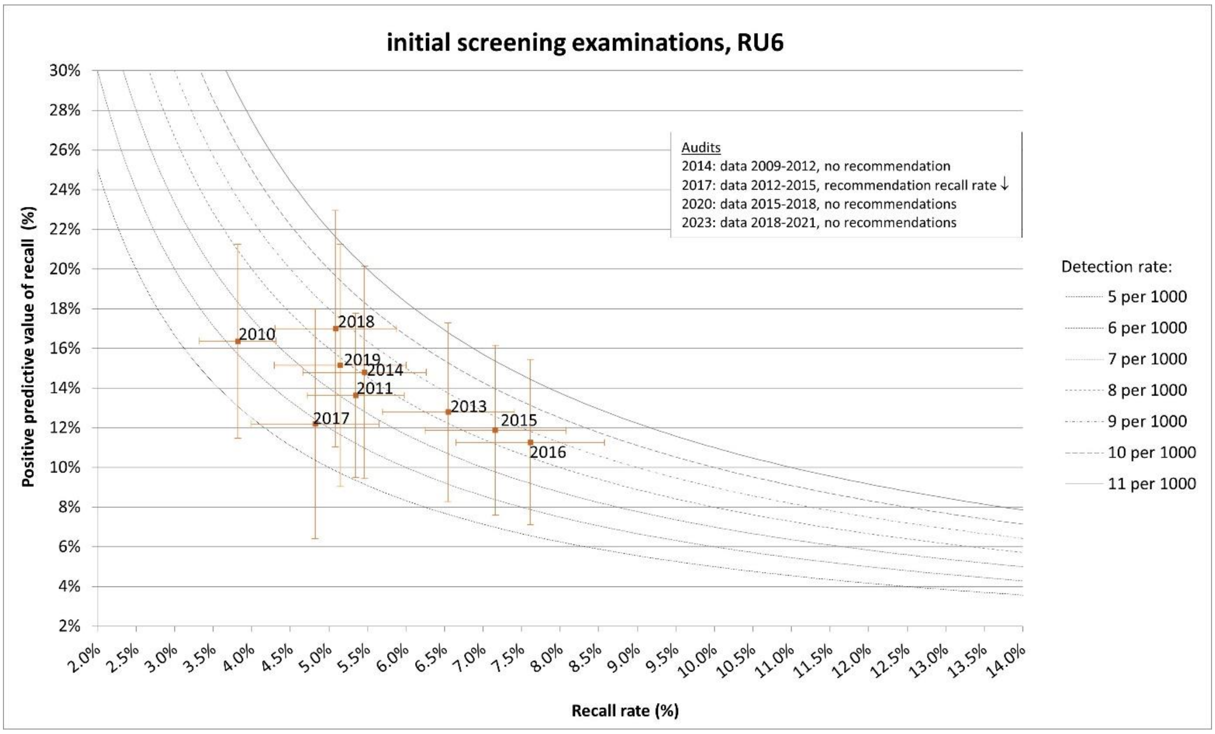

initial screening examinations, RU6
Audits
2014: data 2009-2012, no recommendation
2017: data 2012-2015, recommendation recall rate ↓
2020: data 2015-2018, no recommendations
2023: data 2018-2021, no recommendations
Detection rate:
5 per 1000
6 per 1000
7 per 1000
8 per 1000
9 per 1000
10 per 1000
11 per 1000
Positive predictive value of recall (%)
Recall rate (%)

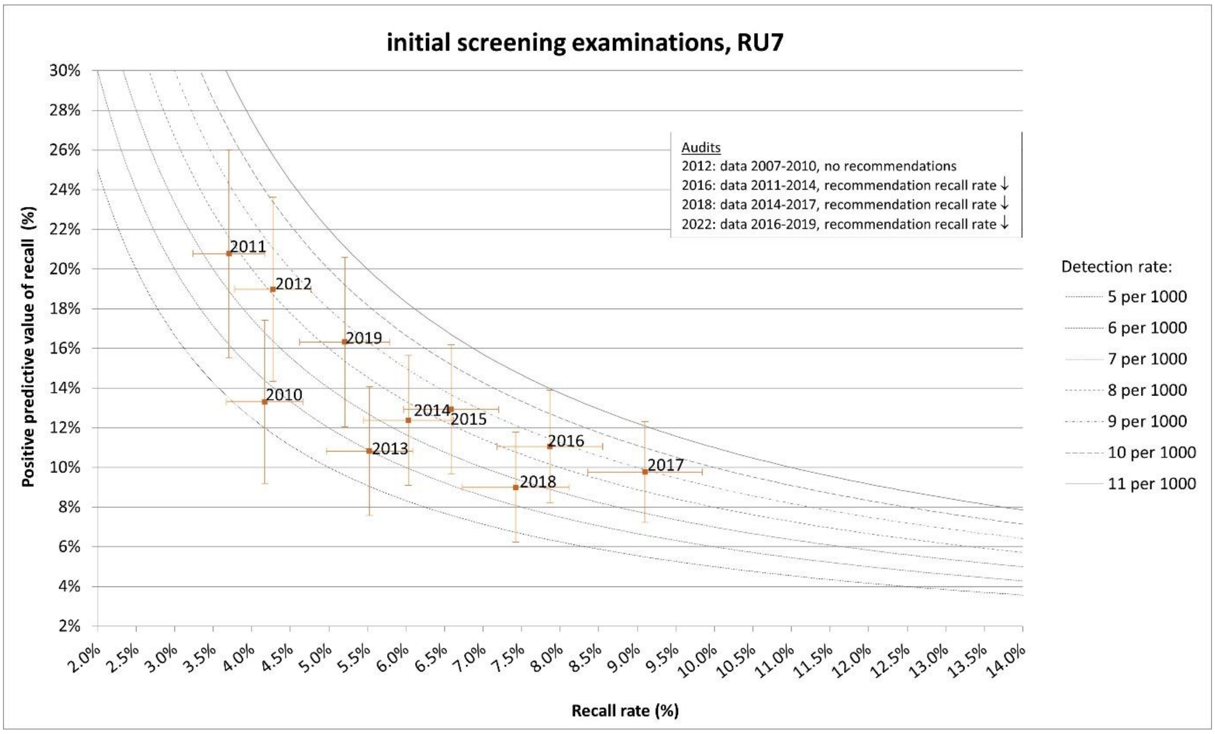

initial screening examinations, RU7
Positive predictive value of recall (%)
Recall rate (%)
Audits
2012: data 2007-2010, no recommendations
2016: data 2011-2014, recommendation recall rate ↓
2018: data 2014-2017, recommendation recall rate ↓
2022: data 2016-2019, recommendation recall rate ↓
Detection rate:
5 per 1000
6 per 1000
7 per 1000
8 per 1000
9 per 1000
10 per 1000
11 per 1000
2010
2011
2012
2013
2014
2015
2016
2017
2018
2019

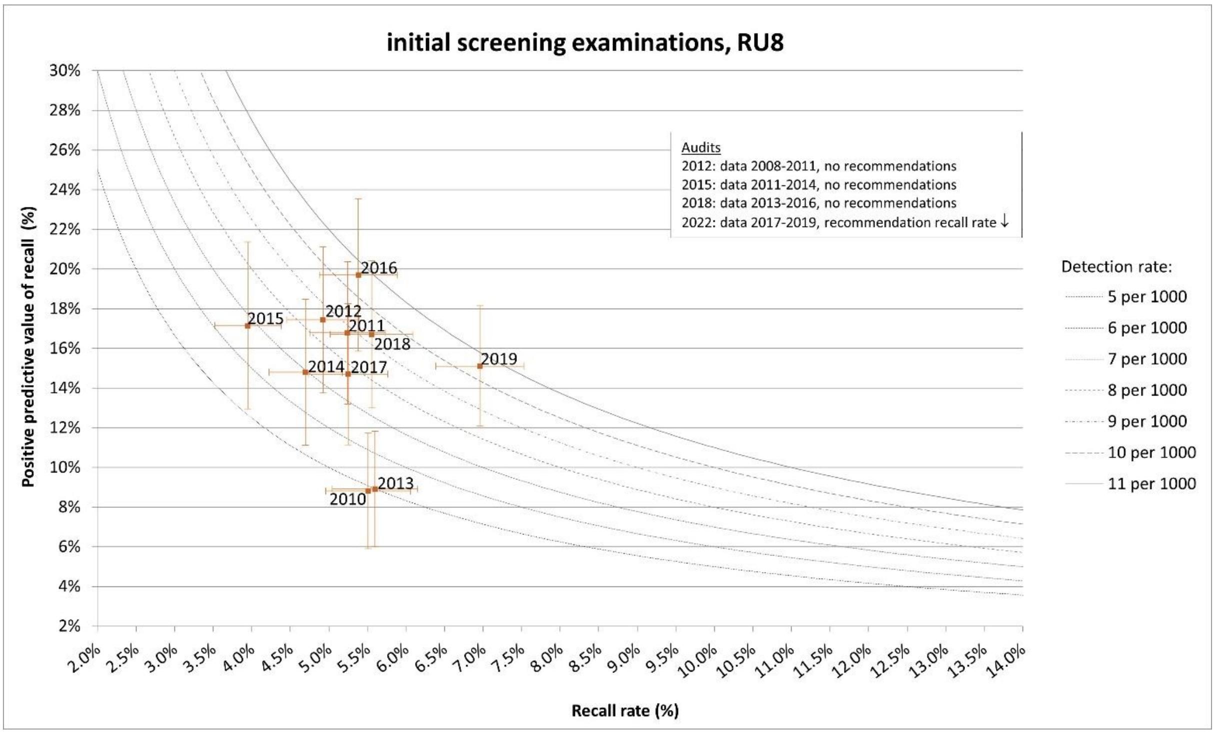

initial screening examinations, RU8
Positive predictive value of recall (%)
Recall rate (%)
Audits
2012: data 2008-2011, no recommendations
2015: data 2011-2014, no recommendations
2018: data 2013-2016, no recommendations
2022: data 2017-2019, recommendation recall rate ↓
Detection rate:
5 per 1000
6 per 1000
7 per 1000
8 per 1000
9 per 1000
10 per 1000
11 per 1000
2010
2011
2012
2013
2014
2015
2016
2017
2018
2019

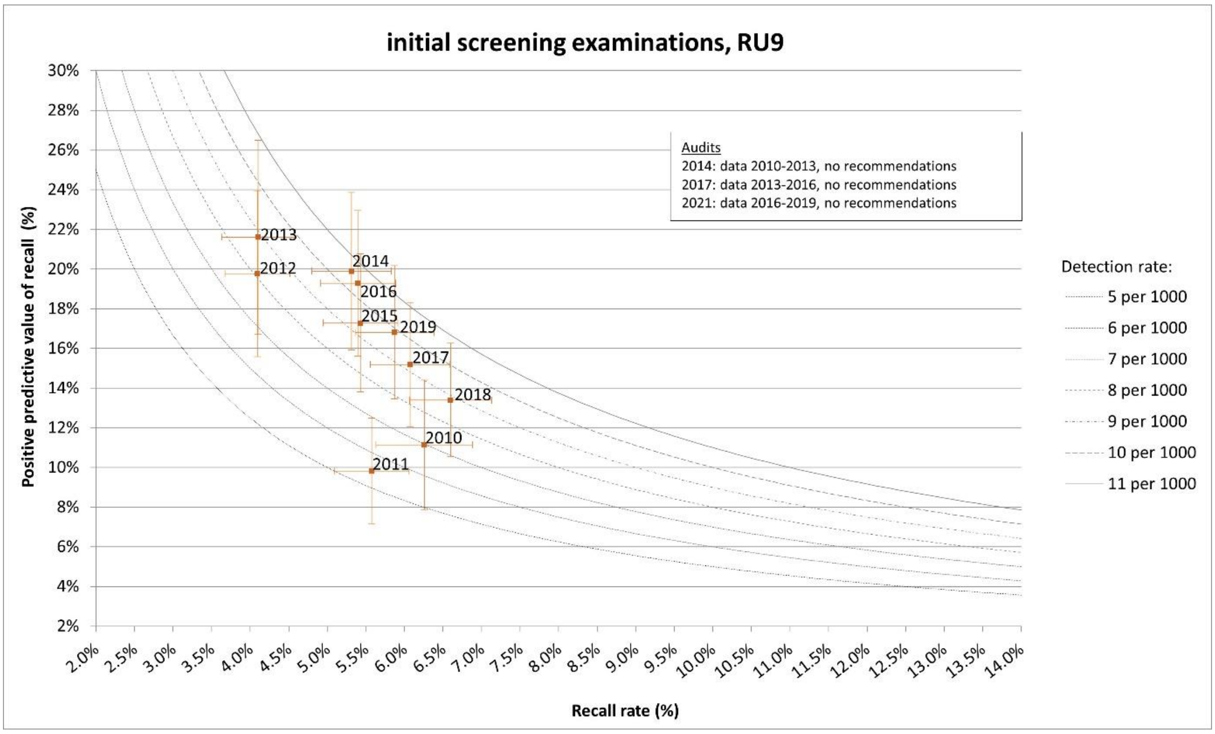

initial screening examinations, RU9
Audits
2014: data 2010-2013, no recommendations
2017: data 2013-2016, no recommendations
2021: data 2016-2019, no recommendations
Detection rate:
5 per 1000
6 per 1000
7 per 1000
8 per 1000
9 per 1000
10 per 1000
11 per 1000
Positive predictive value of recall (%)
Recall rate (%)

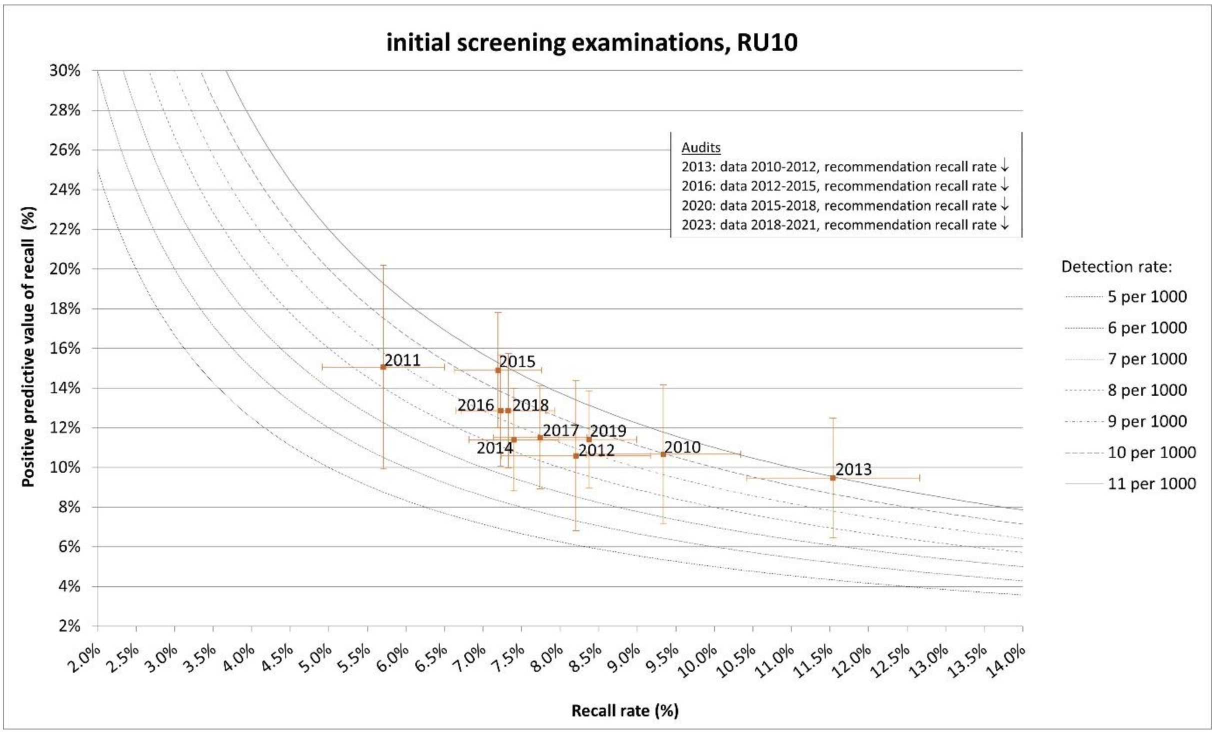

initial screening examinations, RU10
Audits
2013: data 2010-2012, recommendation recall rate ↓
2016: data 2012-2015, recommendation recall rate ↓
2020: data 2015-2018, recommendation recall rate ↓
2023: data 2018-2021, recommendation recall rate ↓
Detection rate:
5 per 1000
6 per 1000
7 per 1000
8 per 1000
9 per 1000
10 per 1000
11 per 1000
Positive predictive value of recall (%)
Recall rate (%)

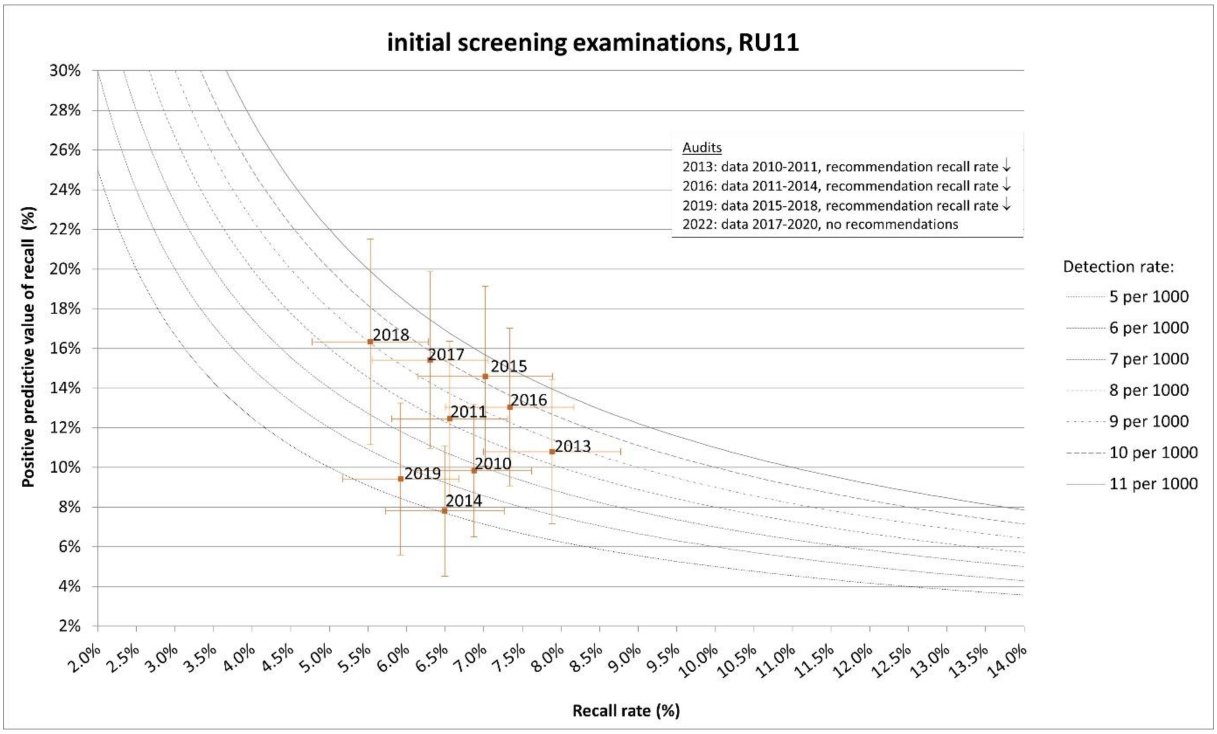
initial screening examinations, RU11
Audits
2013: data 2010-2011, recommendation recall rate ↓
2016: data 2011-2014, recommendation recall rate ↓
2019: data 2015-2018, recommendation recall rate ↓
2022: data 2017-2020, no recommendations
Detection rate:
5 per 1000
6 per 1000
7 per 1000
8 per 1000
9 per 1000
10 per 1000
11 per 1000
2018
2017
2015
2016
2011
2013
2010
2019
2014
Positive predictive value of recall (%)
Recall rate (%)

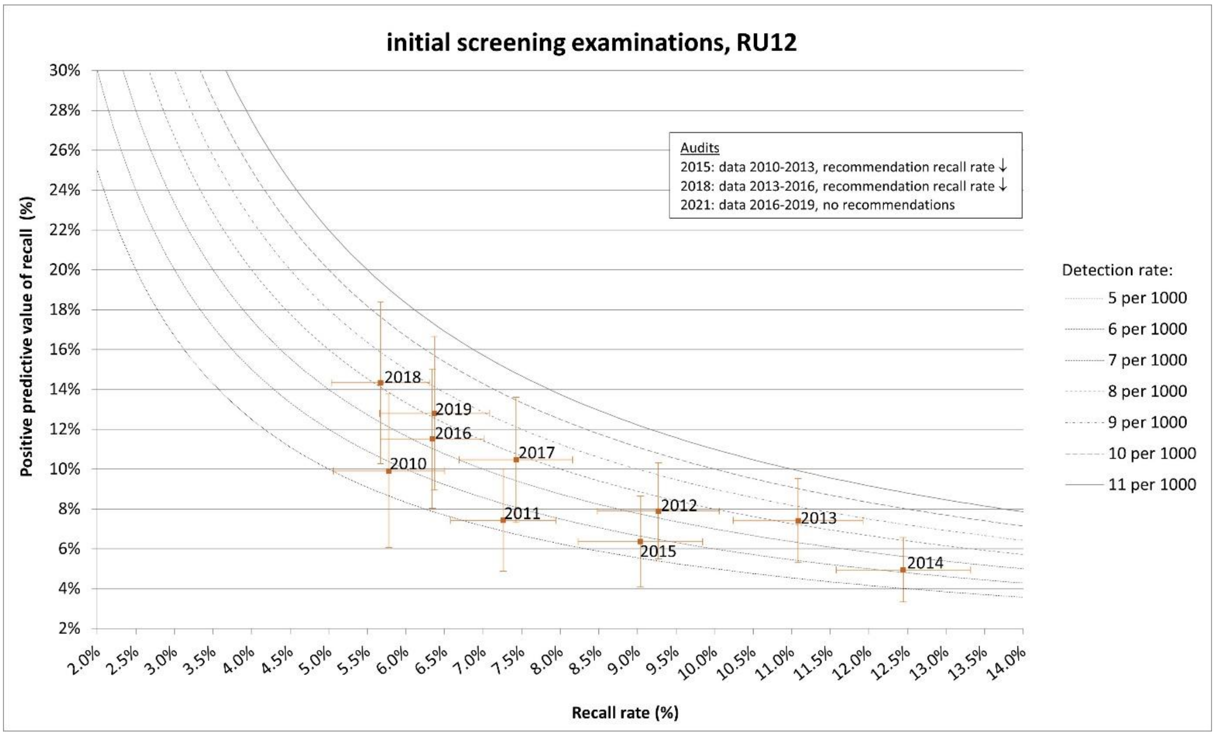
initial screening examinations, RU12
Audits
2015: data 2010-2013, recommendation recall rate ↓
2018: data 2013-2016, recommendation recall rate ↓
2021: data 2016-2019, no recommendations
Detection rate:
5 per 1000
6 per 1000
7 per 1000
8 per 1000
9 per 1000
10 per 1000
11 per 1000
2018
2019
2016
2017
2010
2011
2012
2015
2013
2014
Positive predictive value of recall (%)
Recall rate (%)

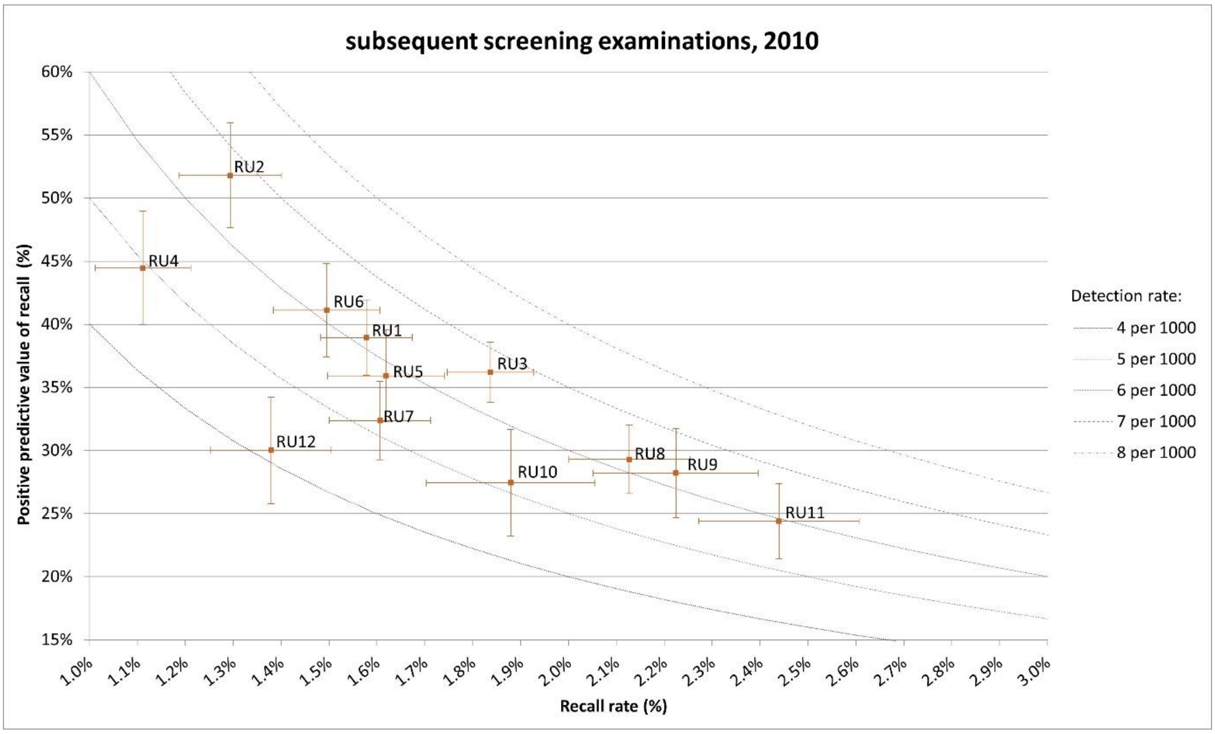
subsequent screening examinations, 2010
Positive predictive value of recall (%)
Recall rate (%)
RU1
RU2
RU3
RU4
RU5
RU6
RU7
RU8
RU9
RU10
RU11
RU12
Detection rate:
4 per 1000
5 per 1000
6 per 1000
7 per 1000
8 per 1000

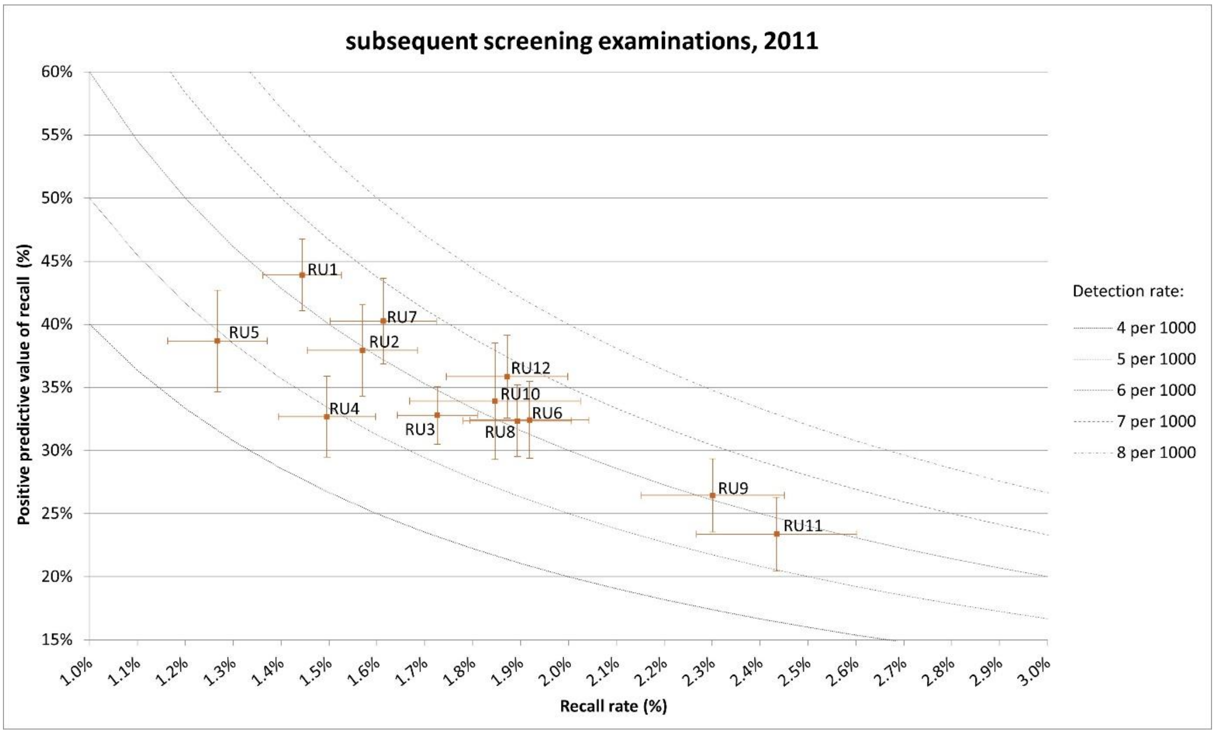
subsequent screening examinations, 2011
Positive predictive value of recall (%)
Recall rate (%)
RU1
RU2
RU3
RU4
RU5
RU6
RU7
RU8
RU9
RU10
RU11
RU12
Detection rate:
4 per 1000
5 per 1000
6 per 1000
7 per 1000
8 per 1000

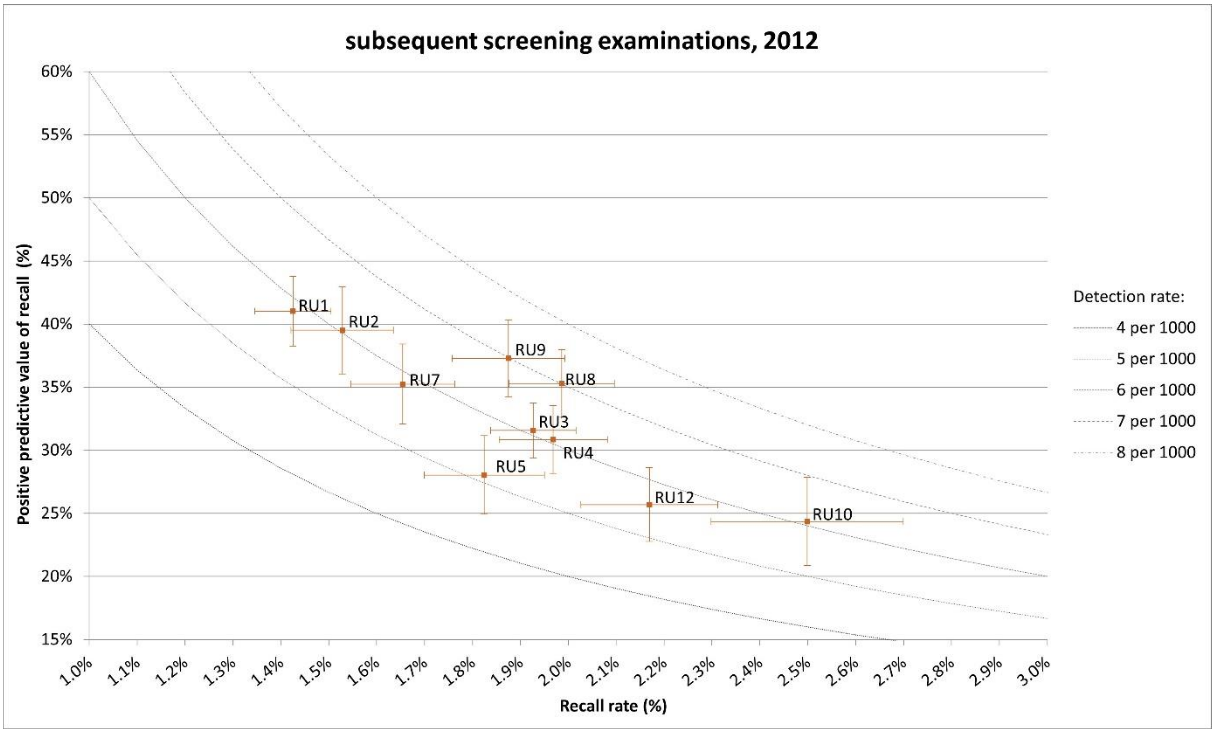
subsequent screening examinations, 2012
Positive predictive value of recall (%)
Recall rate (%)
Detection rate:
4 per 1000
5 per 1000
6 per 1000
7 per 1000
8 per 1000
RU1
RU2
RU7
RU9
RU8
RU3
RU4
RU5
RU12
RU10

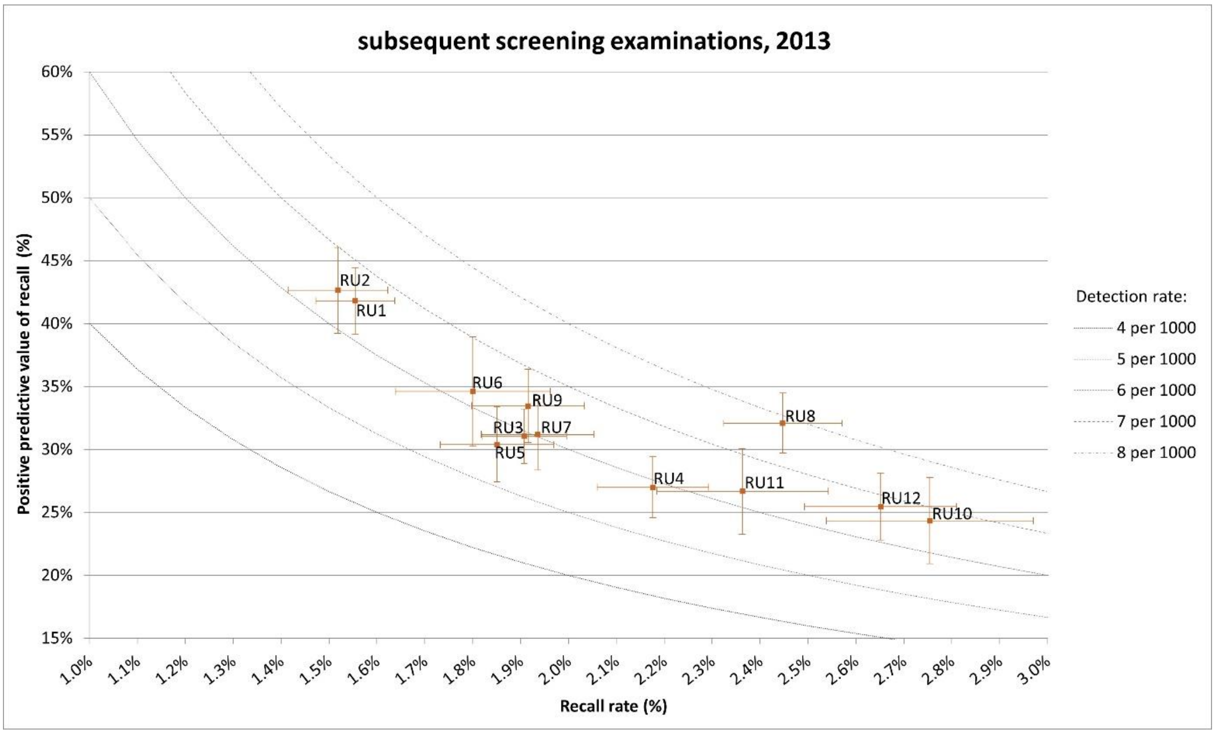
subsequent screening examinations, 2013
Positive predictive value of recall (%)
Recall rate (%)
Detection rate:
4 per 1000
5 per 1000
6 per 1000
7 per 1000
8 per 1000
RU2
RU1
RU6
RU9
RU3
RU7
RU5
RU8
RU4
RU11
RU12
RU10

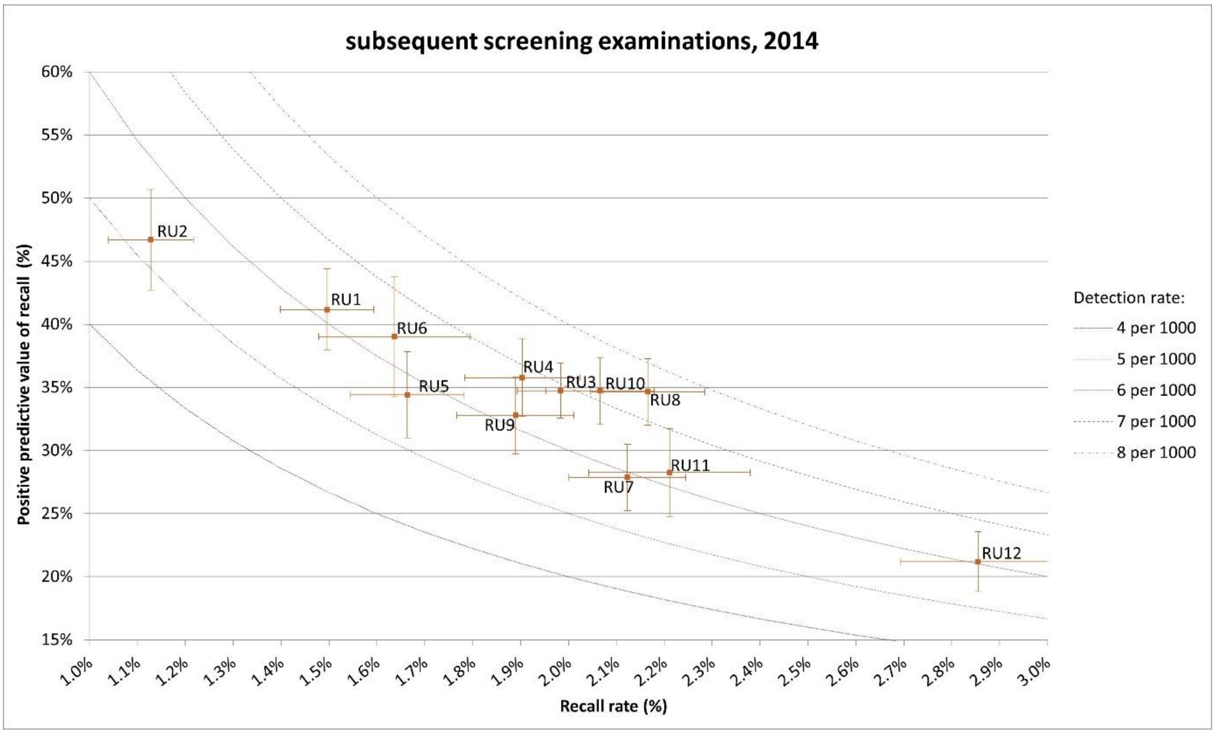
subsequent screening examinations, 2014
Positive predictive value of recall (%)
60%
55%
50%
45%
40%
35%
30%
25%
20%
15%
1.0%
1.1%
1.2%
1.3%
1.4%
1.5%
1.6%
1.7%
1.8%
1.9%
2.0%
2.1%
2.2%
2.3%
2.4%
2.5%
2.6%
2.7%
2.8%
2.9%
3.0%
Recall rate (%)
RU2
RU1
RU6
RU4
RU3
RU10
RU5
RU8
RU9
RU11
RU7
RU12
Detection rate:
4 per 1000
5 per 1000
6 per 1000
7 per 1000
8 per 1000

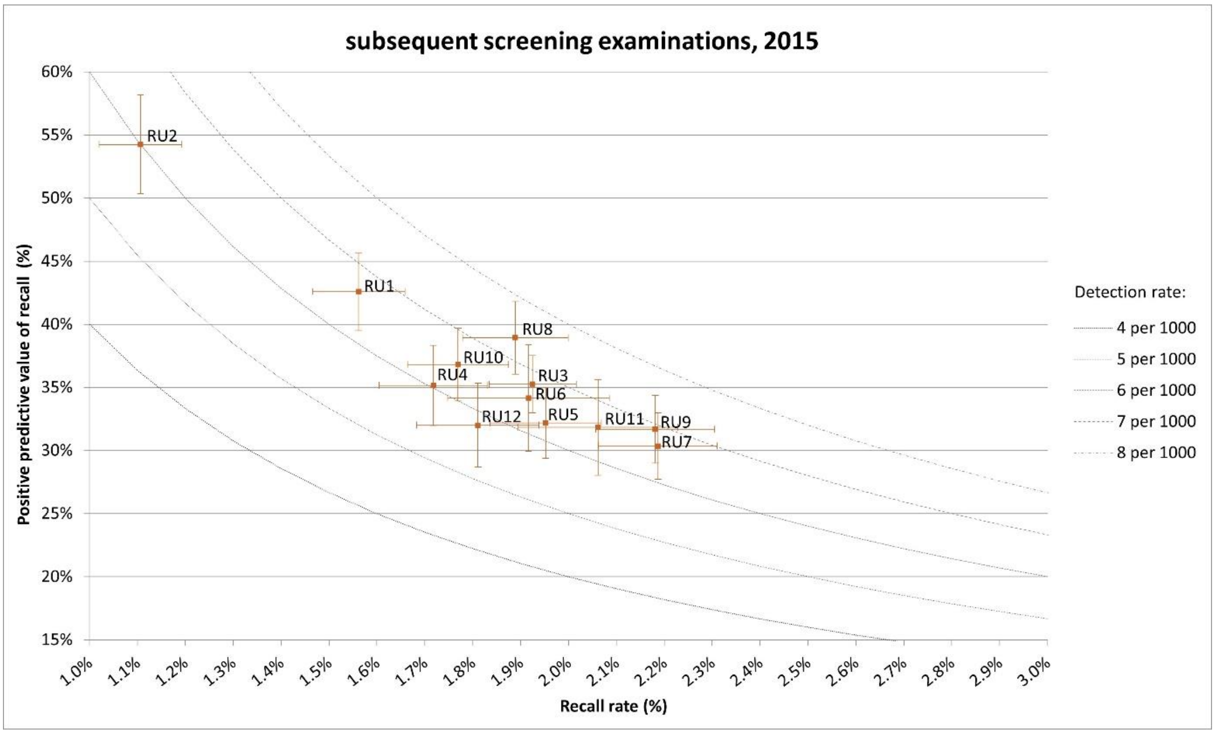
subsequent screening examinations, 2015
Positive predictive value of recall (%)
60%
55%
50%
45%
40%
35%
30%
25%
20%
15%
1.0%
1.1%
1.2%
1.3%
1.4%
1.5%
1.6%
1.7%
1.8%
1.9%
2.0%
2.1%
2.2%
2.3%
2.4%
2.5%
2.6%
2.7%
2.8%
2.9%
3.0%
Recall rate (%)
RU2
RU1
RU8
RU10
RU4
RU3
RU6
RU12
RU5
RU11
RU9
RU7
Detection rate:
4 per 1000
5 per 1000
6 per 1000
7 per 1000
8 per 1000

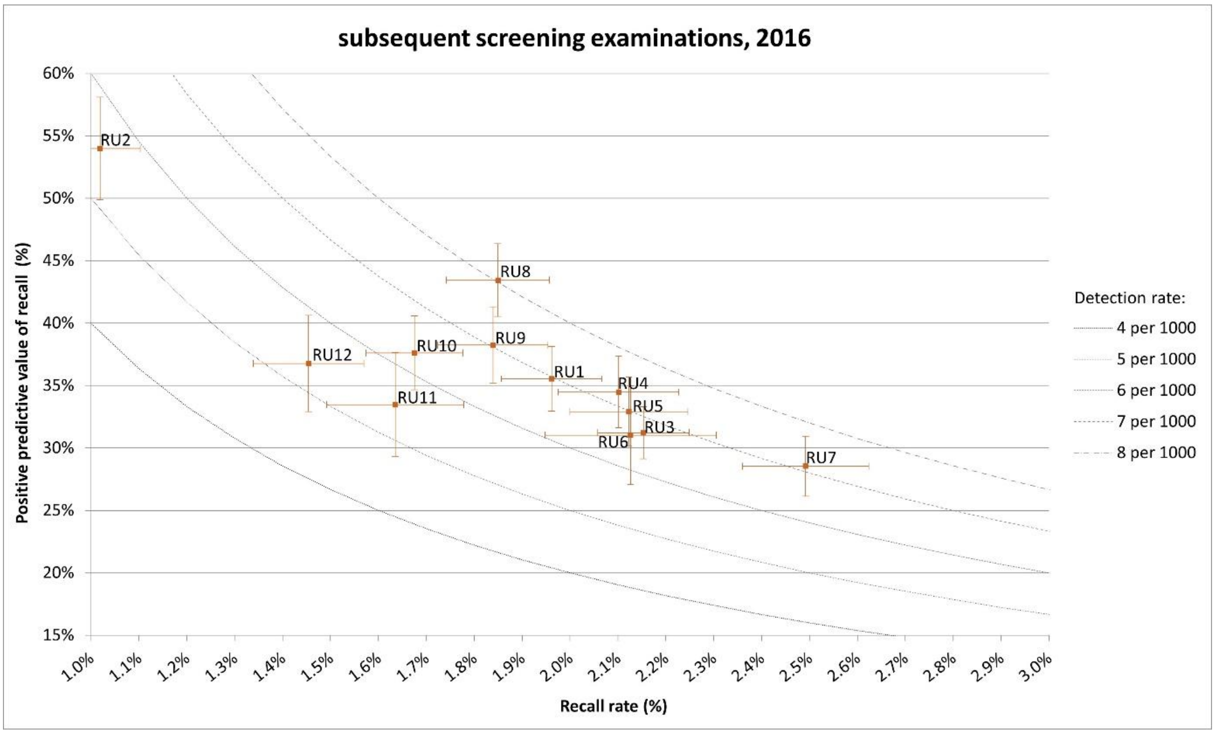
subsequent screening examinations, 2016
Positive predictive value of recall (%)
Recall rate (%)
Detection rate:
4 per 1000
5 per 1000
6 per 1000
7 per 1000
8 per 1000
RU1
RU2
RU3
RU4
RU5
RU6
RU7
RU8
RU9
RU10
RU11
RU12

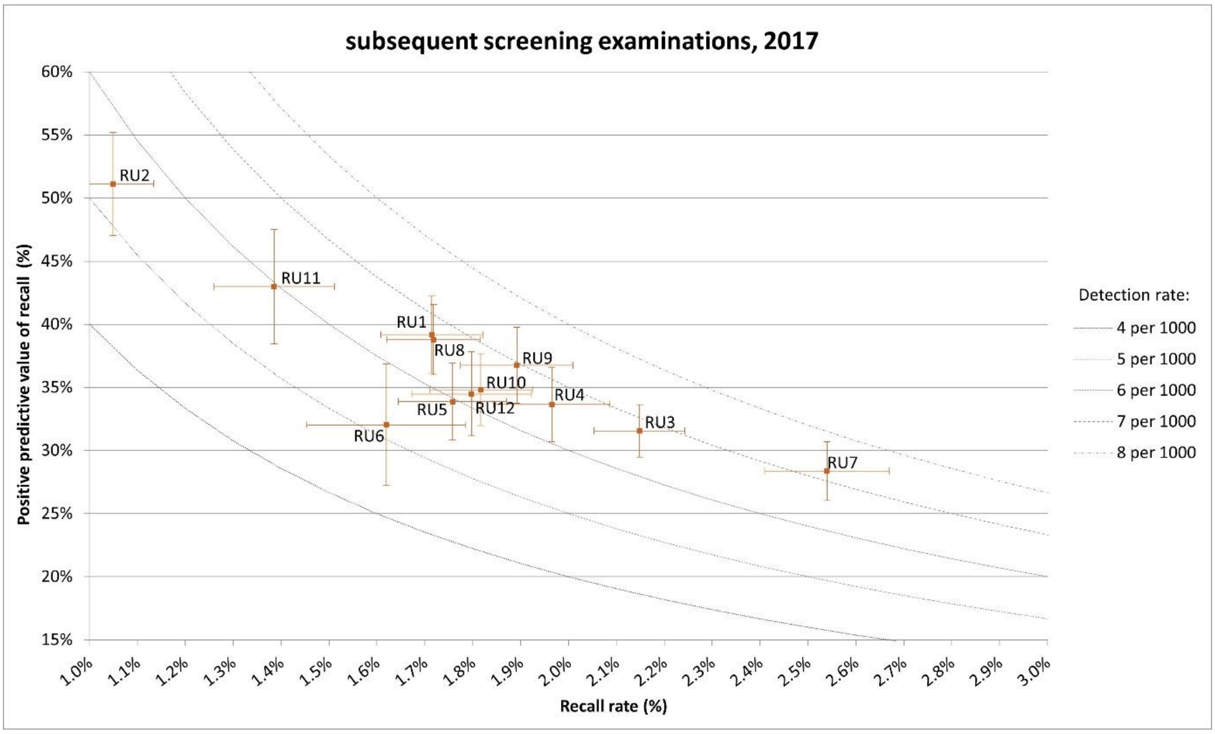
subsequent screening examinations, 2017
Positive predictive value of recall (%)
Recall rate (%)
Detection rate:
4 per 1000
5 per 1000
6 per 1000
7 per 1000
8 per 1000
RU1
RU2
RU3
RU4
RU5
RU6
RU7
RU8
RU9
RU10
RU11
RU12

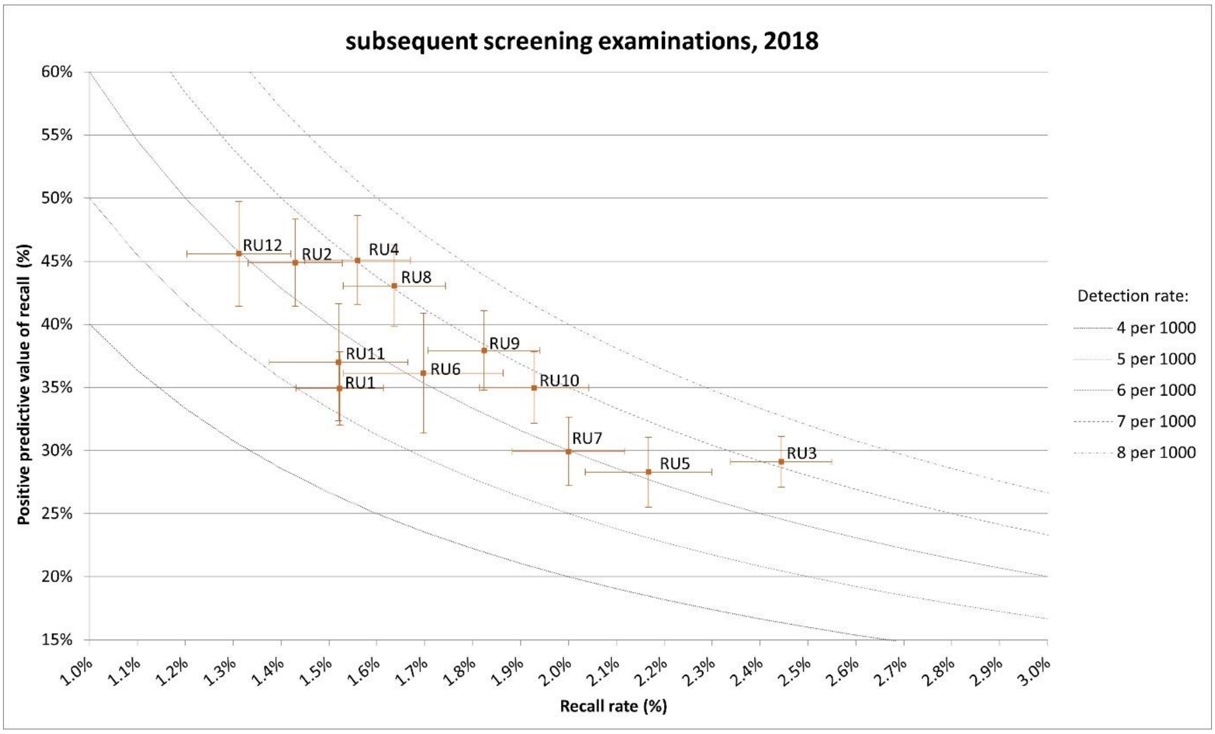
subsequent screening examinations, 2018
Positive predictive value of recall (%)
Recall rate (%)
60%
55%
50%
45%
40%
35%
30%
25%
20%
15%
1.0%
1.1%
1.2%
1.3%
1.4%
1.5%
1.6%
1.7%
1.8%
1.9%
2.0%
2.1%
2.2%
2.3%
2.4%
2.5%
2.6%
2.7%
2.8%
2.9%
3.0%
RU12
RU2
RU4
RU8
RU9
RU11
RU6
RU10
RU1
RU7
RU5
RU3
Detection rate:
4 per 1000
5 per 1000
6 per 1000
7 per 1000
8 per 1000

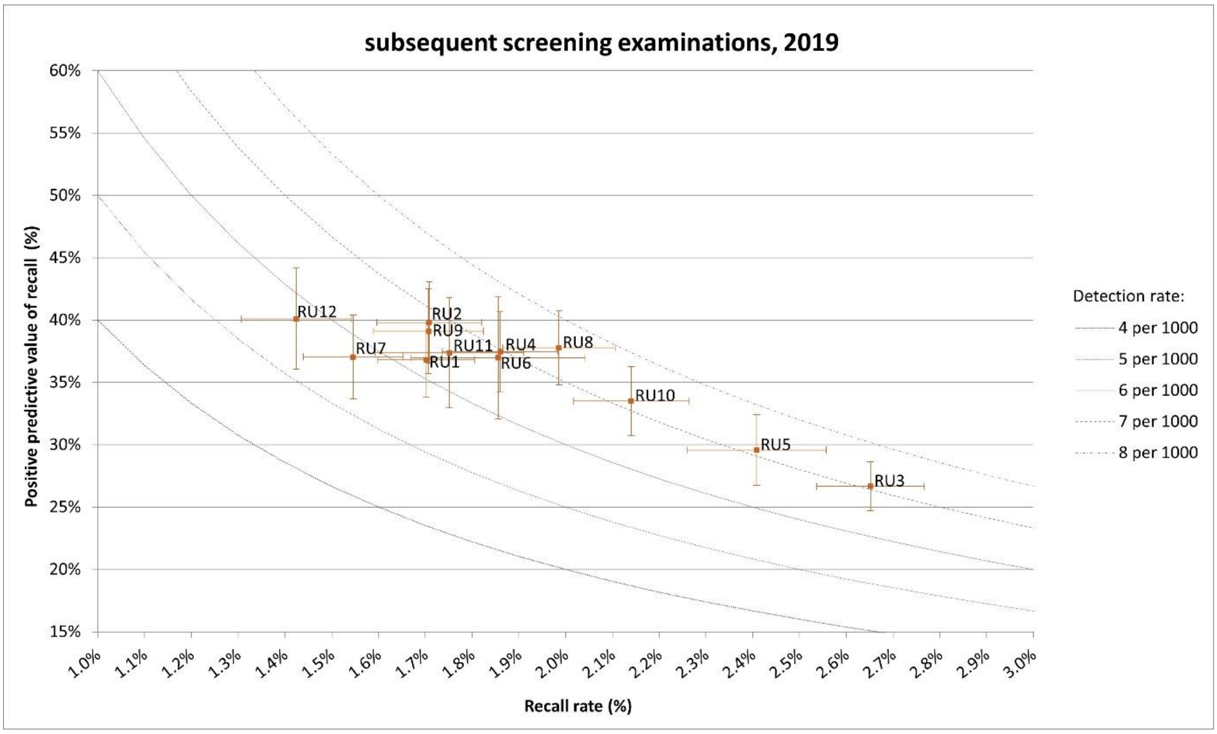
subsequent screening examinations, 2019
Positive predictive value of recall (%)
Recall rate (%)
60%
55%
50%
45%
40%
35%
30%
25%
20%
15%
1.0%
1.1%
1.2%
1.3%
1.4%
1.5%
1.6%
1.7%
1.8%
1.9%
2.0%
2.1%
2.2%
2.3%
2.4%
2.5%
2.6%
2.7%
2.8%
2.9%
3.0%
RU12
RU2
RU9
RU7
RU11
RU4
RU8
RU1
RU6
RU10
RU5
RU3
Detection rate:
4 per 1000
5 per 1000
6 per 1000
7 per 1000
8 per 1000

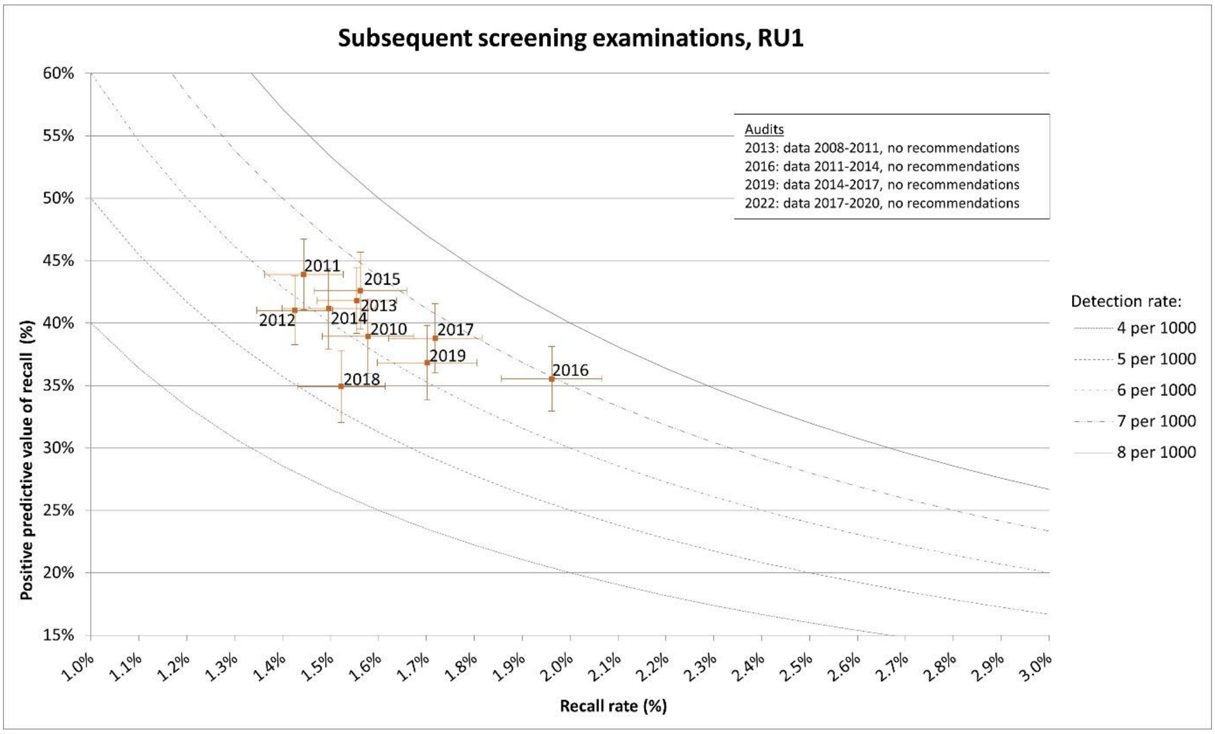
Subsequent screening examinations, RU1
Audits
2013: data 2008-2011, no recommendations
2016: data 2011-2014, no recommendations
2019: data 2014-2017, no recommendations
2022: data 2017-2020, no recommendations
Detection rate:
4 per 1000
5 per 1000
6 per 1000
7 per 1000
8 per 1000
Positive predictive value of recall (%)
Recall rate (%)
2011
2015
2013
2012
2014
2010
2017
2019
2018
2016

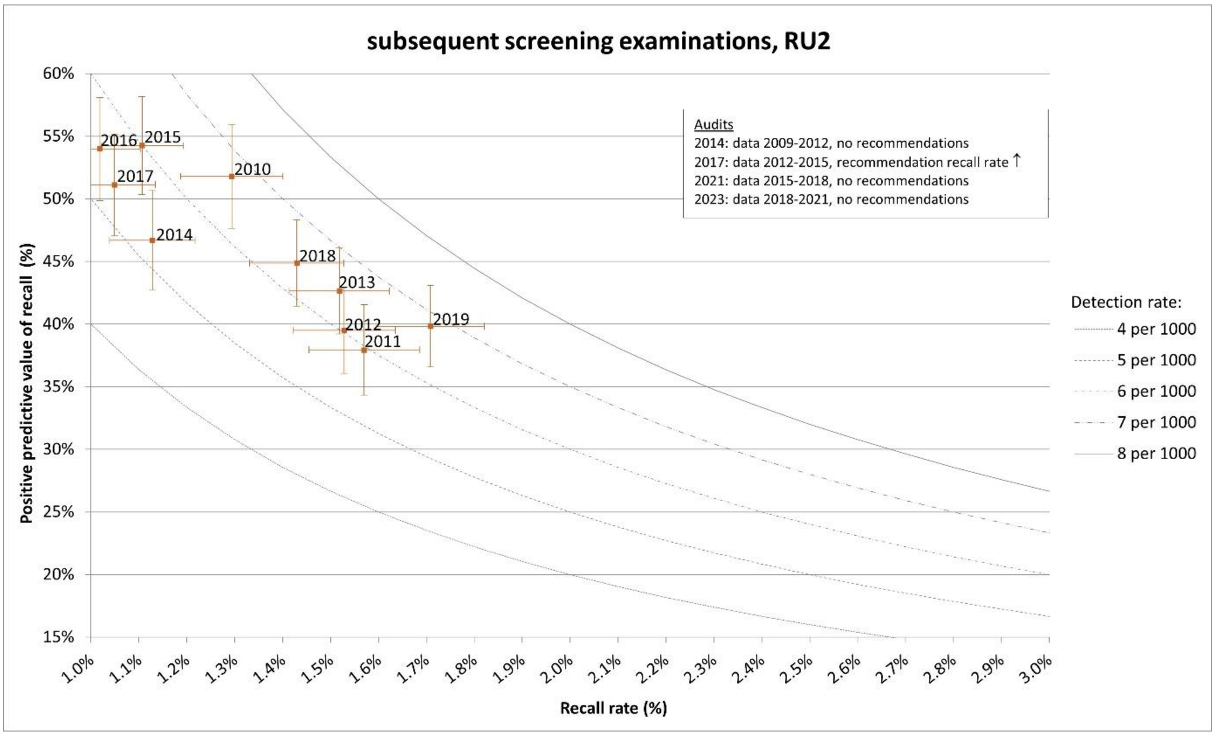
subsequent screening examinations, RU2
Audits
2014: data 2009-2012, no recommendations
2017: data 2012-2015, recommendation recall rate ↑
2021: data 2015-2018, no recommendations
2023: data 2018-2021, no recommendations
Detection rate:
4 per 1000
5 per 1000
6 per 1000
7 per 1000
8 per 1000
Positive predictive value of recall (%)
Recall rate (%)
2016
2015
2017
2010
2014
2018
2013
2019
2012
2011

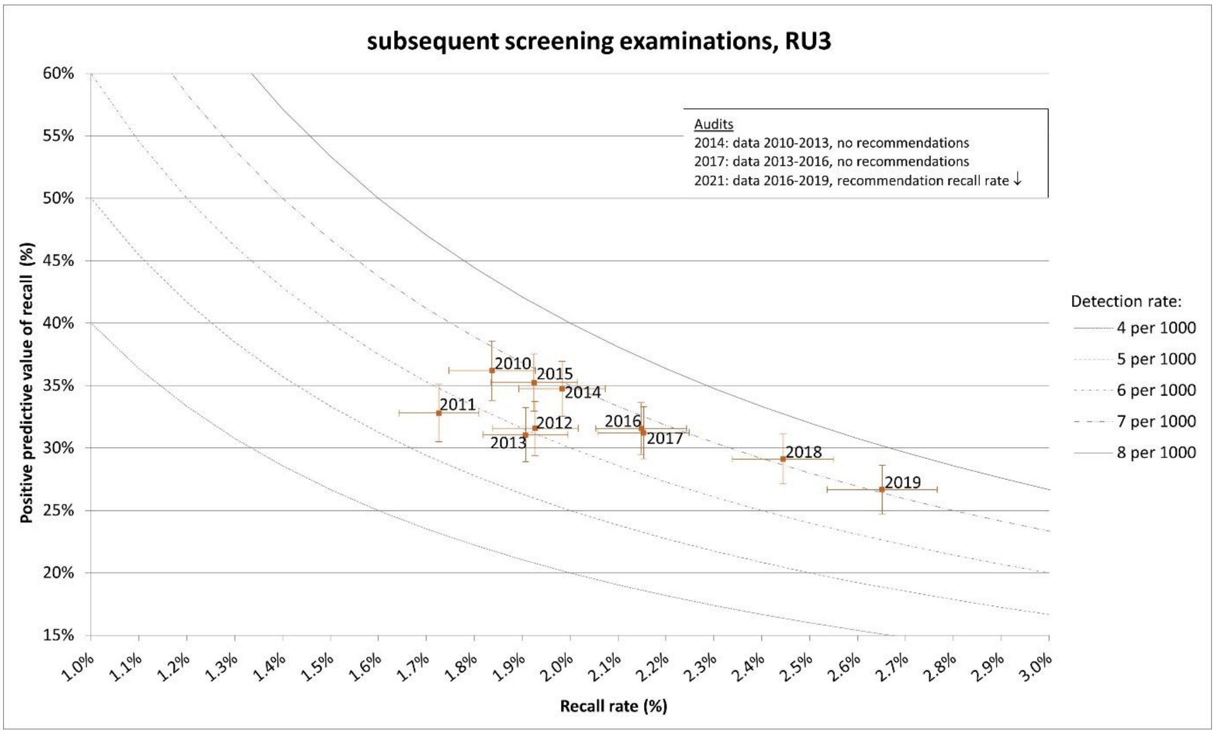

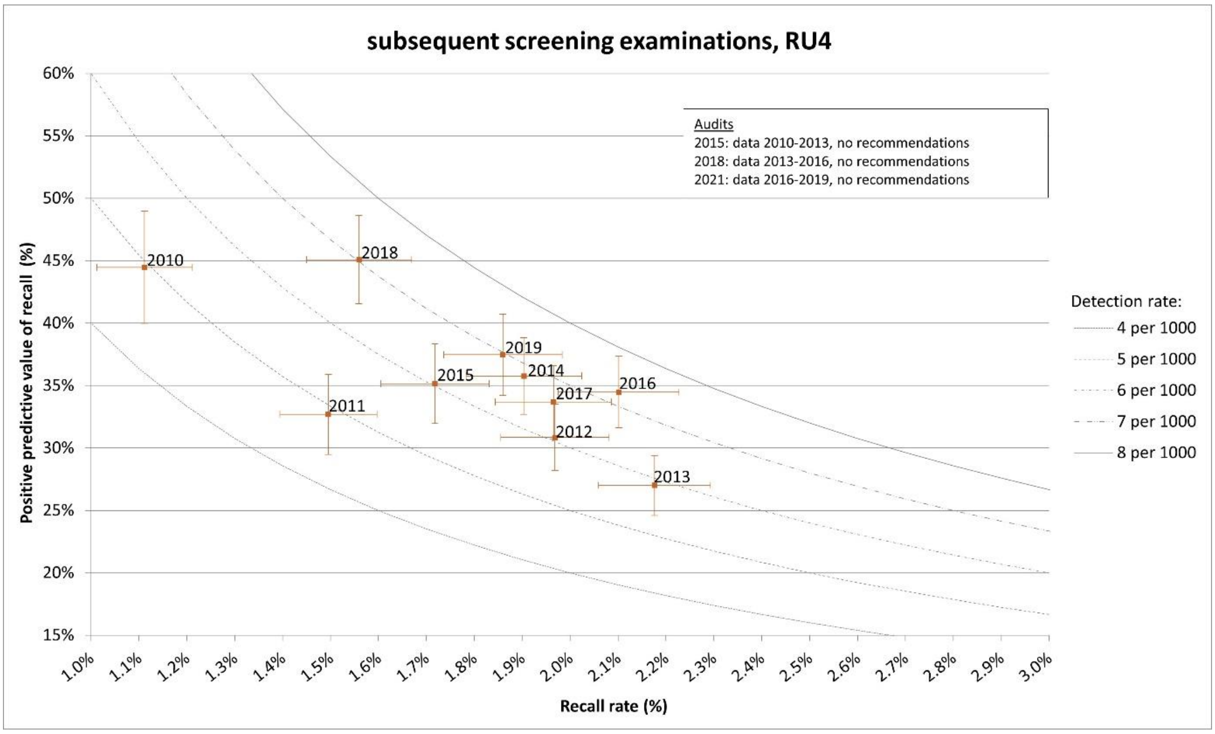

Eur Radiol (2025) Geertse TD, Tetteroo E, Smid-Geirnaerdt MJA, et al

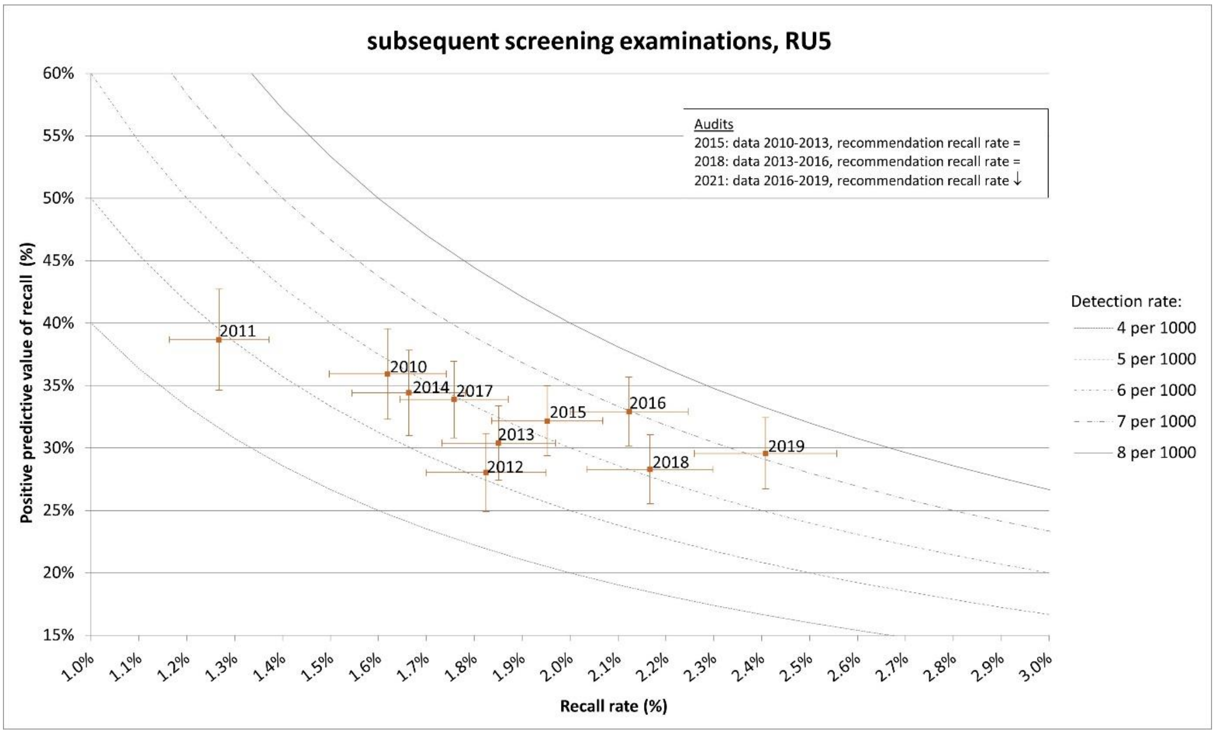
subsequent screening examinations, RU5
Audits
2015: data 2010-2013, recommendation recall rate =
2018: data 2013-2016, recommendation recall rate =
2021: data 2016-2019, recommendation recall rate ↓
Detection rate:
4 per 1000
5 per 1000
6 per 1000
7 per 1000
8 per 1000
Positive predictive value of recall (%)
Recall rate (%)

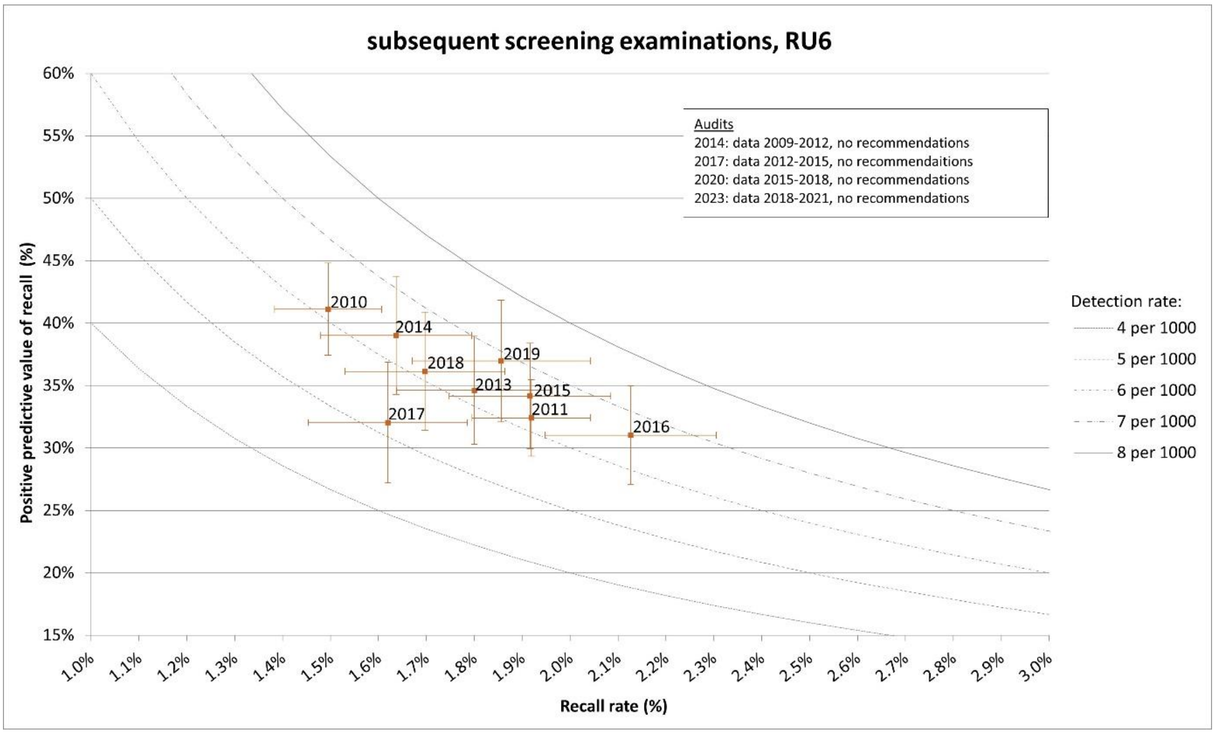
subsequent screening examinations, RU6
Audits
2014: data 2009-2012, no recommendations
2017: data 2012-2015, no recommendaitions
2020: data 2015-2018, no recommendations
2023: data 2018-2021, no recommendations
Detection rate:
4 per 1000
5 per 1000
6 per 1000
7 per 1000
8 per 1000
Positive predictive value of recall (%)
Recall rate (%)

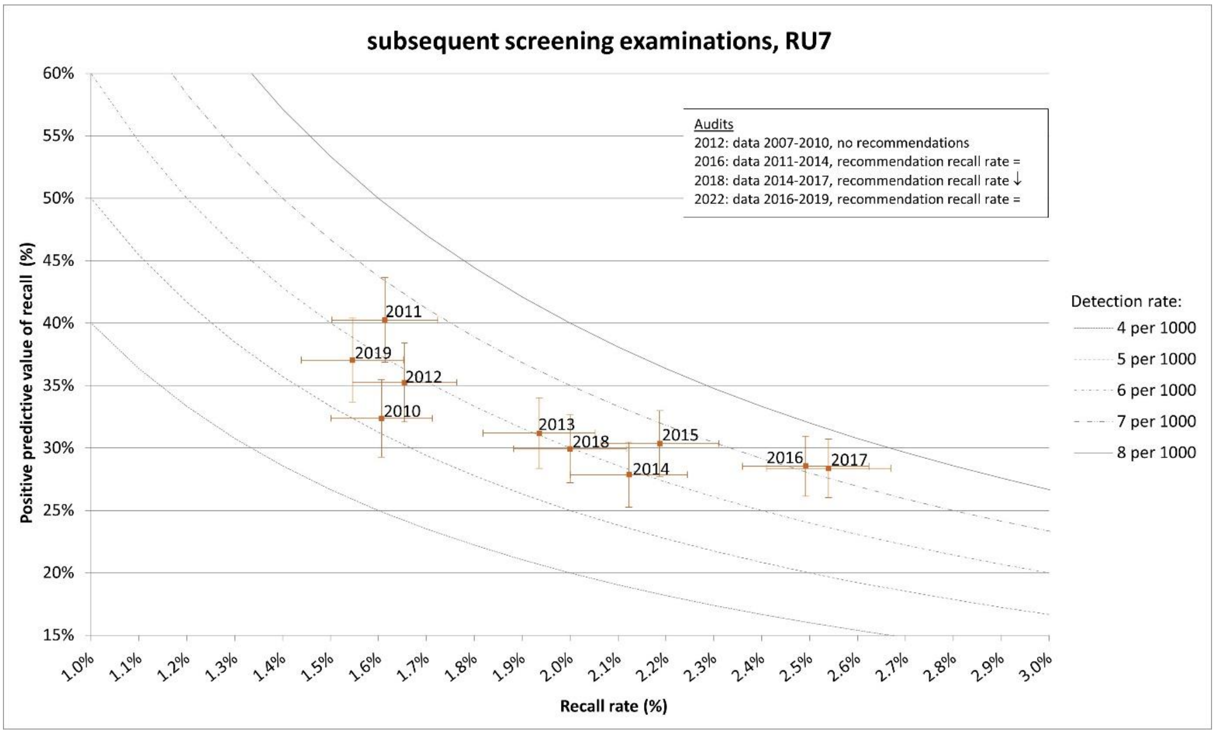

subsequent screening examinations, RU7
Audits
2012: data 2007-2010, no recommendations
2016: data 2011-2014, recommendation recall rate =
2018: data 2014-2017, recommendation recall rate ↓
2022: data 2016-2019, recommendation recall rate =
Detection rate:
4 per 1000
5 per 1000
6 per 1000
7 per 1000
8 per 1000
Positive predictive value of recall (%)
Recall rate (%)
2010
2011
2012
2013
2014
2015
2016
2017
2018
2019

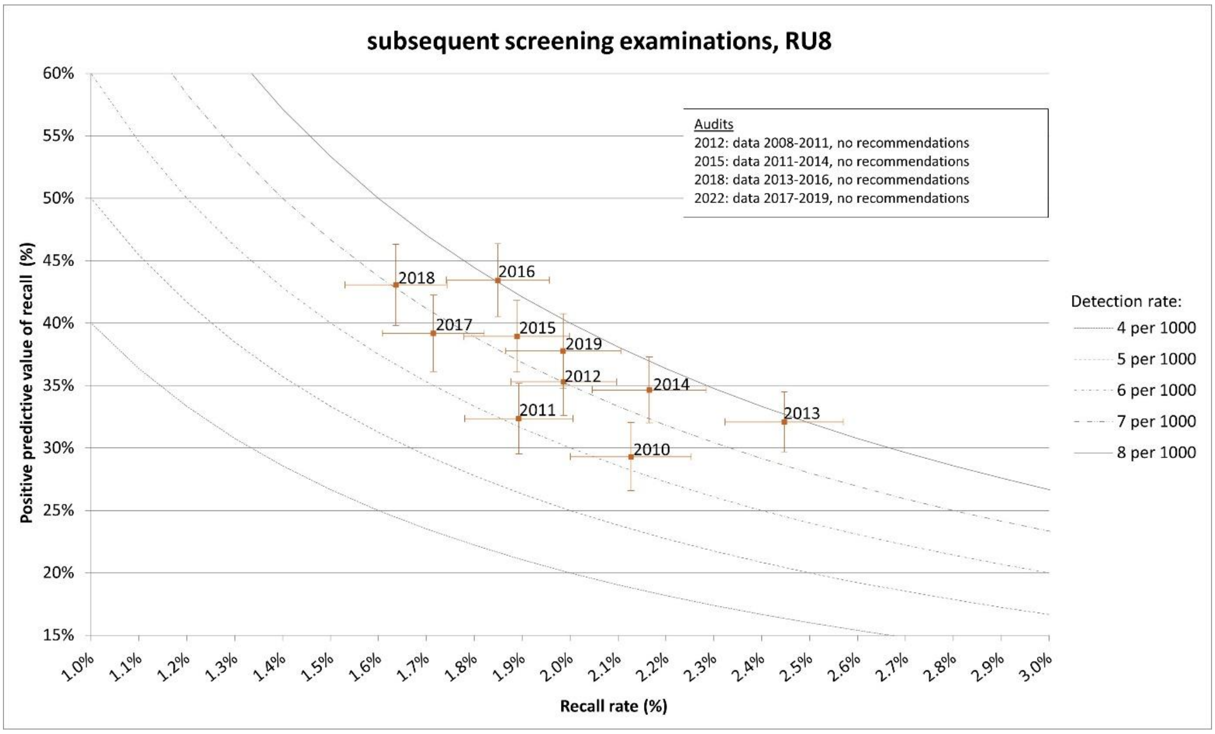

subsequent screening examinations, RU8
Audits
2012: data 2008-2011, no recommendations
2015: data 2011-2014, no recommendations
2018: data 2013-2016, no recommendations
2022: data 2017-2019, no recommendations
Detection rate:
4 per 1000
5 per 1000
6 per 1000
7 per 1000
8 per 1000
Positive predictive value of recall (%)
Recall rate (%)
2010
2011
2012
2013
2014
2015
2016
2017
2018
2019
